# IL-12/23p40 overproduction by dendritic cells leads to an increased Th1 and Th17 polarization in a model of *Yersinia enterocolitica*-induced reactive arthritis in *TNFRp55*^*-/-*^ mice

**DOI:** 10.1371/journal.pone.0193573

**Published:** 2018-03-01

**Authors:** Andrea Constanza Mayordomo, Juan Eduardo Silva, Carolina Virginia Gorlino, José Luis Arias, Walter Berón, María Silvia Di Genaro

**Affiliations:** 1 División de Inmunología, Facultad de Química, Bioquímica y Farmacia, Universidad Nacional de San Luis, San Luis, Argentina; 2 Instituto Multidisciplinario de Investigaciones Biológicas (IMIBIO), Consejo Nacional de Investigaciones Científicas y Técnicas (CONICET), San Luis, Argentina; 3 Instituto de Histología y Embriología, Facultad de Ciencias Médicas, Universidad Nacional de Cuyo - CONICET, Mendoza, Argentina; University of Bergen, NORWAY

## Abstract

Dendritic cells (DCs) play critical functions in the initiation of immune responses. Understanding their role in reactive arthritis (ReA) will help delineate the pathogenesis of this arthropathy. In early studies, we detected IL-12/23p40 deregulation in *Yersinia entercolitica* (Ye)-induced ReA in TNFRp55-deficient (*TNFRp55*^*-/-*^) mice. In this study, we assessed the contribution of DCs in this overproduction. First, greater levels of IL-12/23p40, IFN-γand IL-17A were confirmed in supernatants of lipopolysaccharide (LPS)-stimulated *TNFRp55*^*-/-*^splenocytes obtained on arthritis onset (day 14 after Ye infection). Later, DCs were identified as a precise source of IL-12/23p40 since increased frequency of splenic IL-12/23p40^+^DCs was detected in *TNFRp55*^*-/-*^ mice. After robust *in vivo* amplification of DCs by injection of Fms-like tyrosine kinase 3-Ligand (Flt3L)-transfected BL16 melanoma, DCs were purified. These cells recapitulated the higher production of IL-12/23p40 under TNFRp55deficiency. In agreement with these results, *TNFRp55*^*-/-*^ DCs promoted Th1 and Th17 programs by co-culture with WT CD4^+^lymphocytes. A mechanistic study demonstrated that JNK and p38 MAPK pathways are involved in IL-12/23p40 overproduction in purified *TNFRp55*^*-/-*^ DCs as well as in the JAWS II cell line. This deregulation was once again attributed to TNFRp55 deficiency since CAY10500, a specific inhibitor of this pathway, compromised TNF-mediated IL-12/23p40 control in LPS-stimulated WT DCs. Simultaneously, this inhibition reduced IL-10 production, suggesting its role mediating IL-12/23p40 regulation by TNFRp55 pathway. These results provide experimental data on the existence of a TNFRp55-mediated anti-inflammatory circuit in DCs. Moreover, these cells may be considered as a novel target in the treatment of ReA.

## Introduction

Reactive arthritis (ReA) is a form of seronegative spondyloarthritis (SpA) characterized by predominantly low limb asymmetric oligoarthritis and extra-articular symptoms following gastrointestinal or urogenital infection [1,2]. ReA most commonly affects young adults in the 20–40 year age range, and its incidence ranges between 1–30 cases/100.000 per year [1,3]. The clinical symptoms of ReA manifest 1–3 weeks after infections and in contrast with septic arthritis the blood or synovial cultures are negative. Hence, it has been suggested that ReA is developed by an overstimulated inflammatory response due to bacterial antigens deposited in the joint [4]. However, neither the factors that avoid the complete elimination of these microbial components nor the mechanisms favoring the persistent inflammation are clear.

*Yersinia enterocolitica* (Ye) are Gram-negative bacteria causing food born self-limiting severe diarrhea, enteritis and mesenteric lymphadenitis [5]. In addition to gastrointestinal symptoms, the bacteria eventually disseminate systemically to liver and spleen [6]. Ye serotype O:3 strains are the most frequent cause of human yersiniosis and is a well-established trigger of ReA [7–9]. Cooperative innate and adaptive immune responses including neutrophils, macrophages, T cells, and the cytokines interferon (IFN)-γ, tumor necrosis factor (TNF), interleukin (IL)-12p40 are required to efficiently control Ye infections [10–14]. Although the pathogenesis of ReA after Ye infection is incompletely understood, it has been reported that innate immune reactions, that represent early stages of the host’s response to arthritogenic microbes, may be involved in ReA development [9,15].

TNF is a pleiotropic cytokine considered a major player in the initiation and orchestration of complex events in inflammation and immunity [16]. Upon stimulation by pathogens or inflammatory stimuli, macrophages and T and B lymphocytes are the primary sources of TNF [17]. The biological activities of TNF are mediated by two receptors, TNFRp55 (TNFR1) and TNFRp75 (TNFR2) [18]. TNFRp75 is restricted to specific cell types, such as immune cells, neurons and endothelial cells and it is more efficiently activated by transmembrane TNF, whereas TNFRp55 is ubiquitously expressed and is activated by soluble TNF [16,19]. Furthermore, TNFRp55 is the primary signaling receptor for the majority of the pro-inflammatory and cytotoxic effects classically attributed to TNF [20]. TNFRp55 is recognized as essential for the host immune response to Ye [10,21,22]. In previous studies, we have reported a dual role of TNFRp55: protection from Ye infection in the early immune response, and control of Ye-induced ReA [22,23]. Hence, we found more severe ReA in TNFRp55-deficient (*TNFRp55*^*-/-*^) mice compared with their wild-type (WT) counterparts [23].

The subunit p40 is common for the heterodimeric cytokines IL-12 and IL-23 produced by antigen presenting cells (APCs) in response to certain pathogenic infections. IL-12 promotes Th1 differentiation and IFN-γ production, and IL-23 alternatively, critically contributes to Th17 development and function. IL-12/23p40 deregulation has also been implicated in several autoimmune diseases [24]. Recently, we have reported critical differences of IL-12/23p40 in joint draining lymph nodes of *TNFRp55*^*-/-*^mice indicating the involvement of IL-12/23p40 in the generation of augmented production of IFN-γ and IL-17 in Ye-induced ReA under TNFR p55 deficiency [23,25].

Dendritic cells (DCs) orchestrate immune responses presenting antigens to T lymphocytes and driving their activation and differentiation to effector T cells [26]. DCs may play an important role in the initiation of joint inflammation. Thus, intra-articular injection of collagen-pulsed pro-inflammatory mature DCs is sufficient to initiate arthritis in mice [27]. Furthermore, DCs are believed to be crucial to the pathogenesis and progression of rheumatoid arthritis [28]. It has been reported that TNFRp55 has paradoxical anti-inflammatory effects on IL-12/23p40 expression in macrophages and DCs [29]. However, DC involvement in the onset of ReA remains unclear. Hence, the purpose of this work is to evaluate whether DCs participate in the higher amount of IL-12/23p40in the pathophysiological scenario of Ye-inducedReAin*TNFRp55*^*-/-*^mice. The results of this study uncover new immunopathogenic mechanisms involved in Ye-induced ReA linking DCs and TNFRp55 with Th1 and Th17 responses in this arthritis.

## Materials and methods

### Mice

*TNFRp55*^*-/-*^ mice (C57BL/6) were kindly provided by the Max von Pettenkofer Institute, Munich, Germany. C57BL/6 WT mice were purchased from the Animal Facilities of the National University of La Plata, Argentina. Breeding colonies were established at the Animal Facilities of the National University of San Luis, Argentina. Male mice aged between 8 to10 weeks old were used. Mice were kept under specific pathogen-free conditions and provided with sterile food and water *ad libitum*.

### Ethics statement

Experimental protocols were approved by the Animal Care and Use Committee of the National University of San Luis, Argentina (Approved protocol number: B 169/13). The condition of the animals was monitored daily. All efforts were made to minimize suffering or distress.

### Bacterial culture and infection

Ye O:3, strain MHC700 (kindly provided by Dr. G. Kapperud, Department of Bacteriology, Oslo, Norway) was used for infection. Bacteria were cultured as described earlier [21]. Mice were starved for 3h before and after orogastric infection with 1-5x10^8^yersiniae in 0.2 ml PBS using a gastric tube. The number of inoculated bacteria was controlled by plating of serial dilutions of the inoculated suspension on Trypticase Soy Agar (TSA) and counting the colony forming units (CFU) number after incubation at 27°C for 48h.

### *In vivo* expansion and isolation of DCs

Female *TNFRp55*^*-/-*^ or WT mice (4–5 per group) were subcutaneously injected with 5–8 x 10^6^ Flt3L-transfected BL16 melanoma [30] (gently provided by Dr. Gabriel Morón, Universidad Nacional de Córdoba, Argentina) in 200 μl saline. When tumors were palpable in both mice groups, DCs were isolated from the spleen by magnetic beads using MACS anti-mouse CD11c particles according to manufacturer´s instructions (MiltenyiBiotec Inc. CA, USA). The purity of DCs CD11c^+^ (> 95%) was evaluated by flow cytometry.

### *In vitro* cultures

On day 14 after infection, splenocytes were obtained as described previously [23]. Cells were seeded onto 24-well plates (2 x 10^6^ cells/well) and cultured for 24h at 37°C under an atmosphere of 5% CO_2_ in DMEM medium (Life Technologies, CA, USA) supplemented with 10% fetal bovine serum (FBS), 2 mM L-glutamine, 1 mM pyruvate, 100 IU/ml penicillin and 100 μg/ml streptomycin. The cells were stimulated with 1 μg/ml of LPS (*Escherichia coli* 0111:B4, Sigma, St Louis, MO, USA) or incubated with medium alone. Culture supernatants were separated from the cells at different times and stored at -20°C until cytokine determinations. After 24h, the cells were harvested and analyzed by flow cytometry.

The JAWS II cell line [31] was gently provided by Dr. Ignacio Cebrian, (Universidad Nacional de Cuyo, Argentina). These cells were grown in RPMI-1640 media (Life Technologies) supplemented with 10% FCS, 2 mM L-glutamine, 1 mM pyruvate, 100 U/ml penicillin,100μg/ml streptomycin, and 5 ng/ml of recombinant granulocyte/macrophage-colony stimulating factor (GM-CSF) (Penprotech, Rocky Hill, NJ; US). For stimulation, the JAWS II cells were treated with1 μg/ml of LPS alone or in combination with 60 ng/ml of hTNF (Miltenyi Biotec Inc) for 24h.

For the study of MAPK or TNFR involvements, cells were treated with JNK (SP600125), ERK (PD98059), or p38 MAPK (SB203580) inhibitors (10 μM, all from Calbiochem, San Diego, CA, US). One hour after treatment with these inhibitors, cells were stimulated with LPS for 24h. Etanercept (1ng/ml, Enbrel; Pfizer, NY, US) was used for TNF inhibition through both TNFRp55 and TNFRp75, and CAY10500 (1ng/ml, Santa Cruz, CA, US) for specific inhibition of TNFRp55 during 24h of stimulation with LPS alone or in combination with hTNF. Supernatants were collected and stored at -20°C until cytokine determinations.

### Cytokine quantification

TNF, IL-12/IL-23p40, IL-17A, IFN-γ and IL-10 levels were determined in cell culture supernatants using Enzyme-linked immunosorbent assay (ELISA) kits according to the manufacturer’s instructions. TNF, IFN-γ and IL-10 kits were obtained from eBioscience (San Diego, CA, USA), IL-17A kit from Biolegend (San Diego, CA, USA), and IL-12/23p40 kit from BD Biosciences (San Diego, CA, USA).

### Cell preparation and flow cytometry

Antibodies were all purchased from BD Biosciences, unless indicated otherwise. Briefly, cells were first incubated with anti-mouse CD16/32 (Fc block) for 15 min at 4°C and then stained with FITC, PE, APC or PerCP.Cy5.5 conjugates of anti-MHC-II (clone 2G9), anti-CD11c (clone HL3, Biolegend), anti-F4/80 (clone CI:A3-1), anti-CD80 (clone 16-10A1), anti-CD86 (clone GL1) or anti-CD11b (clone M1/70) for 30 min at 4°C. For unconjugated anti-CD120a (TNFRp55, clone 55R-286) or anti-CD120b (TNFRp75, clone TR75-89) antibodies, the cells were stained a second time with PE-conjugated anti-hamster polyclonal antibody.

For intracellular staining of splenocytes, the cells were stimulated with PMA/ionomycin (50/750 ng/ml, eBioscience) for 5 h in the presence of GolgiPlug (BD Biosciences). After washing, cells were stained with anti-MHC-II and anti-CD11c, fixed with 1% paraformaldehyde, and then a permeabilizing solution BD FACS (BD Biosciences) was applied for intracellular staining with anti-IL-12/23p40 (clone C15.6).

For detection of Th1 and Th17 lymphocytes, the cells were stained with anti-CD4 (clone RM4-5) and anti-CD3 (clone 145-2C11), fixed and permeabilized as described above using anti-IL-17A (clone eBio17B7C15.6, eBioscience) or anti-IFN-γ (clone XMG1.2).

Data were obtained on a FACSCalibur flow cytometer (BD Biosciences) and analyzed with FlowJo software (Tree Star, Ashland, OR).

### Co-culture of DC and T-cells

On day 5 after Ye infection, CD4^+^ T cells were enriched from the spleen of *TNFRp55*^*-/-*^ or WT mice by magnetic beads using BD IMag anti-mouse CD4 particles according to manufacturer’s instructions (BD Biosciences). Cell population purity (> 95%) was checked by flow cytometry analysis.

*In vivo* expanded and isolated splenic DCs from both mouse groups were *in vitro* infected with Ye (moi: 10:1) for 1h, and treated afterwards with gentamicin (100 μg/ml,Life Technologies) for 24h at 37°C to kill extracellular bacteria. Then, they were washed and co-cultured with the WT or *TNFRp55*^*-/-*^ CD4^+^ T-cells at a 1:10 ratio in 96-well culture plates for 5 days at 37 °C under an atmosphere of 5% CO_2_. The IFN-γ and IL-17A levels were quantified in the supernatants by ELISA, and Th1 and Th17 cells were analyzed by flow cytometry as described above.

### Statistical analysis

Statistical significance was assessed by Student T-test, one-way analysis of variance (ANOVA) or two-way ANOVA followed by Tukey or Bonferroni multiple comparison test as appropriate. All experiments were performed at least twice. A *p* value ≤ 0.05 was considered statistically significant. Data were analyzed using GraphPad Prism 5.0 software (GraphPad Software, La Jolla, CA, USA).

## Results

### TNFRp55 deficiency and pro-inflammatory cytokine profile in Ye-infected spleen cells

**Fig 1:**
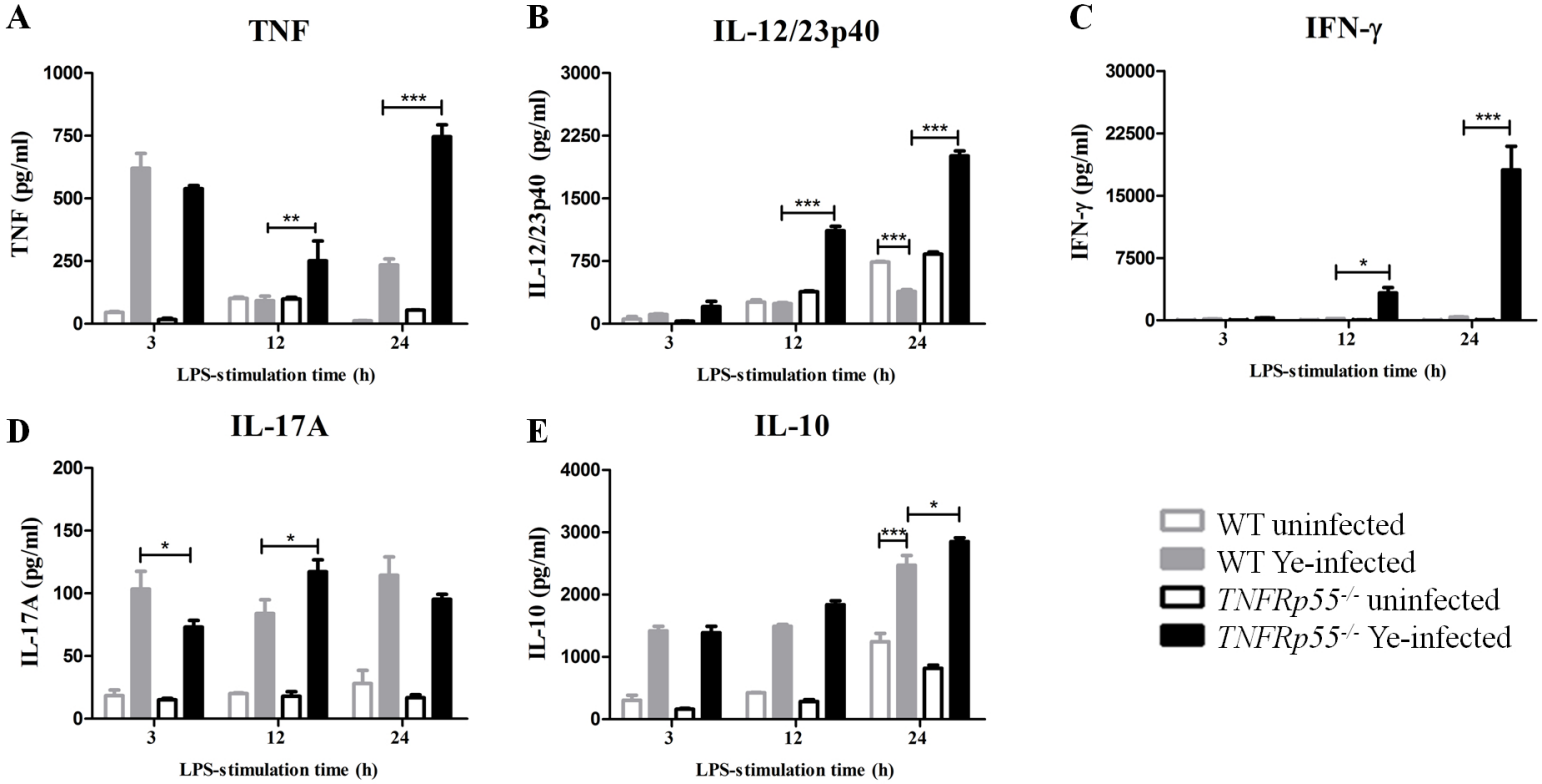
Cytokine secretion by LPS-stimulated WT and *TNFRp55*^*-/-*^ splenocytes on ReA onset. Splenic cells were obtained from WT and *TNFRp55*^*-/-*^mice on day 14 after Ye infection (ReA onset), and stimulated *in vitro* with LPS. Splenocytes from uninfected mice (PBS) were used as controls. After 3, 12 and 24h of stimulation, supernatants were collected and assayed by ELISA for TNF (A), IL-12/23p40 (B), IFN-γ (C), IL-17A (D) and IL-10 (E). Values represent the mean ± SEM of quadruplets at each time point of one out of two independent experiments, with five mice per group. *P<0.05; ** *P*<0.01; *** *P*<0.001.

*TNFRp55*^*-/-*^ mice are susceptible to orogastric Ye O:3 infection [22], and those that survive the infection develop chronic ReA whose onset is on day 14 [23]. To examine the cytokine profile of spleen cells of *TNFRp55*^*-/-*^ on ReA onset, first we obtained splenocytes from *TNFRp55*^*-/-*^ and WT mice on day 14 after the Ye infection. At the same time, cells from both groups of mice receiving phosphate-buffered saline (PBS) were used as controls. We assessed the cytokines secreted by the cells at different times after *in vitro* stimulation with LPS, and found early TNF production by cells of infected mice (Fig 1A). Splenocytes from infected *TNFRp55*^*-/-*^mice produced significantly higher TNF, IL-12/23p40 and IFN-γ levels compared with splenocytes from infected WT mice (Fig 1A–1C). Although cells from both groups of infected mice secreted elevated levels of IL-17A, after 12h of LPS-stimulation, supernatants of infected *TNFRp55*^*-/-*^splenocytes reached the highest IL-17 amounts (Fig 1D).

Interestingly, when we analyzed the levels of the anti-inflammatory cytokine IL-10, a significant amount of IL-10 was produced by infected splenocytes from both *TNFRp55*^*-/-*^ and WT mice. However, the IL-10 levels appear to be insufficient to control IL-12/23p40 secretion under TNFRp55 deficiency (Fig 1E and 1B). Higher IL-10 secretion by infected versus uninfected WT cells at 24h of stimulation (Fig 1E), seems to efficiently regulate IL-12/23p40 production by infected WT splenocytes resulting lower levels than PBS cells of WT mice (Fig 1B). Thus, the absence of TNFRp55 on spleen cells from infected mice compromises the control of IL-12/23p40 production under inflammatory stimulation.

### TNFRp55-deficient DCs as cellular sources of the IL-12/23p40 subunit

**Fig 2:**
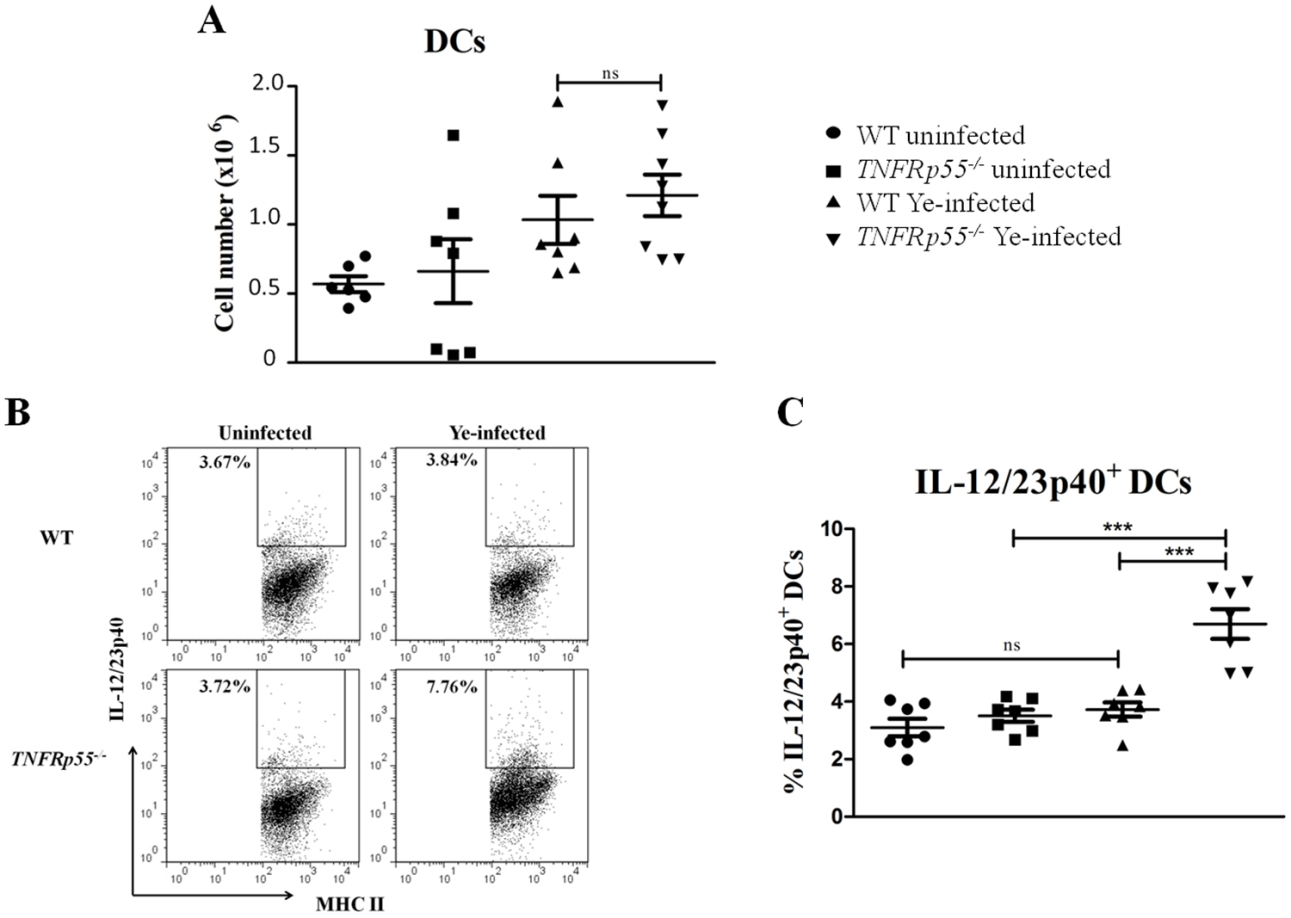
IL-12/23p40^+^ dendritic cells in spleen of WT and *TNFRp55*^*-/-*^ mice on ReA onset. Splenic cells were obtained from WT and *TNFRp55*^*-/-*^ mice on day 14 after Ye infection (ReA onset). Dendritic cells (DCs) were stained for cell surface CD11c and MHC-II, and analyzed by flow cytometry. (A) Absolute DC number in the spleen of Ye-infected WT and *TNFRp55*^*-/-*^ mice are presented. Splenic DCs of uninfected (PBS) mice were used as controls. (B) Representative dot plot showing analysis of IL-12/23p40^+^ DCs (CD11c^+^MHC-II^+^ gate) in splenocytes from control (PBS), and Ye-infected WT and *TNFRp55*^*-/-*^ mice. The cells were stimulated with PMA/Ionomycin and brefeldin for 5 h, and then stained for cell surface CD11c and MHC-II, and intracellular IL-12/23p40, and then analyzed by flow cytometry. The numbers in the plots indicate the percentages of labeled cells in representative mice. (C) Percentage of IL-12/23p40^+^ DCs of the sum of three independent experiments. Each symbol represents an individual mouse; horizontal lines indicate the mean ± SEM. ***P<0.001. ns: not significant.

**Fig 3:**
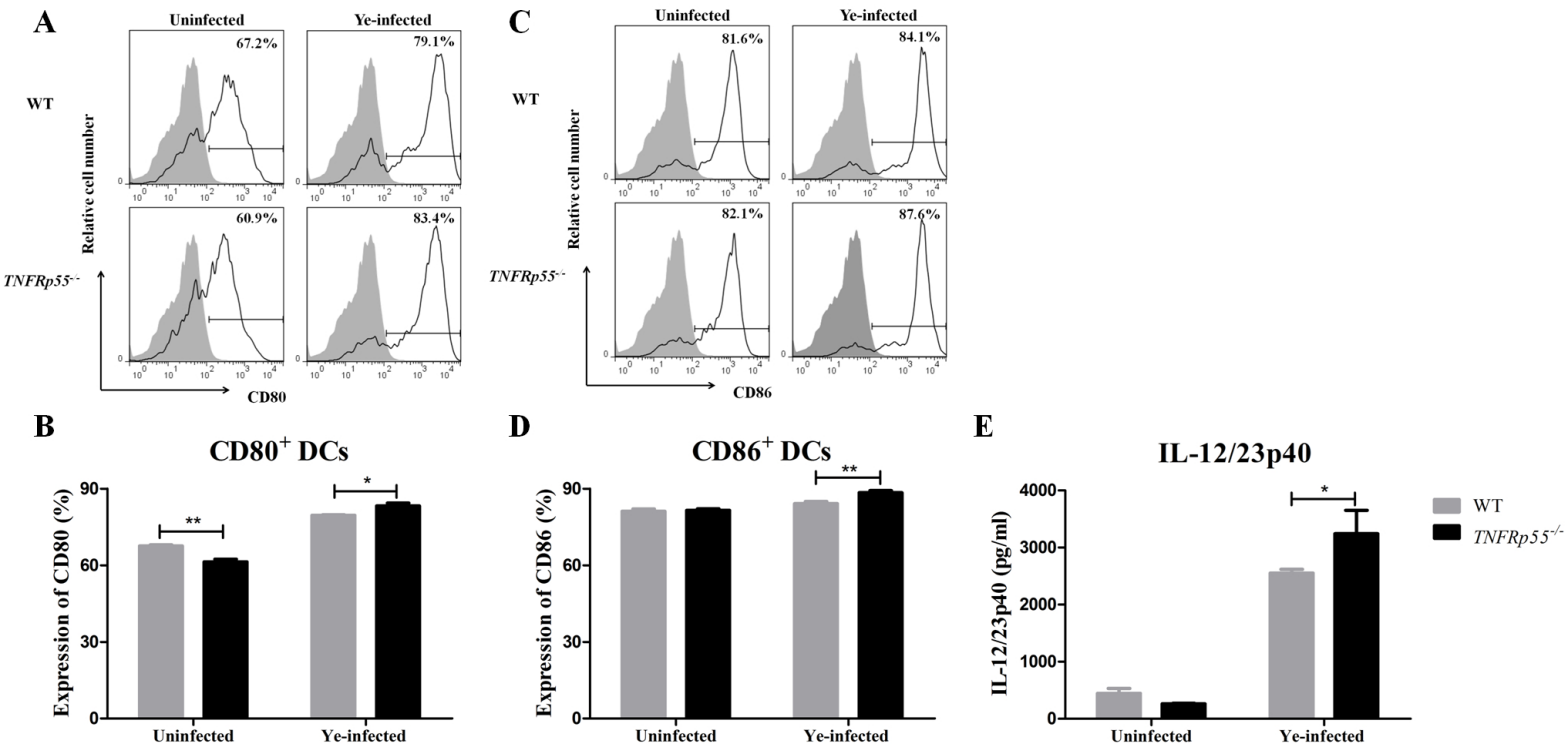
Activation profile of and IL-12/23p40 secretion by isolated dendritic cells. Dendritic cells (DCs) were expanded *in vivo* in WT and *TNFRp55*^*-/-*^ mice. The cells were isolated and infected *in vitro* with Ye. Representative overlaid flow cytometry histogram analysis showing CD80 (A) and CD86 (C) expressions of uninfected and Ye-infected splenic DCs. Percentages of DCs CD80^+^(B) and CD86^+^ (D), and IL-12/23p40 secretion in the supernatants (E) expressed as the mean ± SEM of quadruplets of one out of two independent experiments with four mice per group. *P<0.05; ** *P*<0.01.

To evaluate the cellular sources of IL-12/23p40 observed after LPS-stimulation, the number of DCs in spleen cells of *TNFRp55*^*-/-*^ and WT mice obtained on day 14 after Ye infection was assessed. No significant differences were found when the number of splenic DCs was compared between *TNFRp55*^*-/-*^ and WT mice (Fig 2A, and S1 Fig). However, the frequency of splenic *TNFRp55*^*-/ -*^IL-12/23p40^+^ DCs was significantly higher as compared to WT DCs (Fig 2B and 2C, and S1 Fig). Thus, DCs of TNFRp55-deficient mice are important cellular sources for the overproduction of IL-12/23p40 detected on Ye-induced ReA onset in *TNFRp55*^*-/-*^ mice. To determine the role of TNFRp55 in DCs on the control of IL-12/23p40, experiments with isolated DCs were carried out. Although DCs are central APC, they are relatively scarce *in vivo* compared with other leukocyte populations. Therefore, since Flt3L has been identified as the primary differentiation factor for DCs [30,32], we expanded DCs by injection of Flt3L-transfected BL16 melanoma in *TNFRp55*^*-/-*^ and WT mice to generate an experimentally useful amount of DCs. Then, the activation phenotype of isolated splenic*TNFRp55*^*-/-*^ DCs were compared with WT DCs after *in vitro* infection with Ye O:3. Although probably activated by the purification process, CD80 and CD86 expressions in Ye-infected *TNFRp55*^*-/-*^ DCs were fairly higher than WT DCs, indicating increased activation in the absence of TNFRp55 (Fig 3A–3D, and S2 Fig). In addition, the frequency of IL-12/23p40^+^ DCs isolated from spleen of *TNFRp55*^*-/-*^mice was again significantly higher compared with those cells of WT mice (Fig 3E). Thus, deficiency of TNFRp55 signaling increases activation and IL-12/23p40 production in DCs under *in vitro* Ye infection.

### *TNFRp55*^*-/-*^ DCs promoting Th1 and Th17 differentiation of CD4^+^ lymphocytes

IL-12/23p40 subunit is shared by IL-12 and IL-23, which are cytokines that encourage Th1 and Th17 differentiation, respectively [24]. As described before, high frequency of *TNFRp55*^*-/-*^ IL-12/23p40^+^ DCs was found (Fig 2B and 2C). As a functional assay for DCs, we then assessed Th1 and Th17 frequencies in co-culture experiments. Therefore, DCs were isolated from the spleen of *TNFRp55*^*-/-*^ and WT mice and infected *in vitro* with Ye. These cells were co-cultured with CD4^+^lymphocytes purified from *TNFRp55*^*-/-*^ or WT mice on day 5 after Ye-infection.

**Fig 4:**
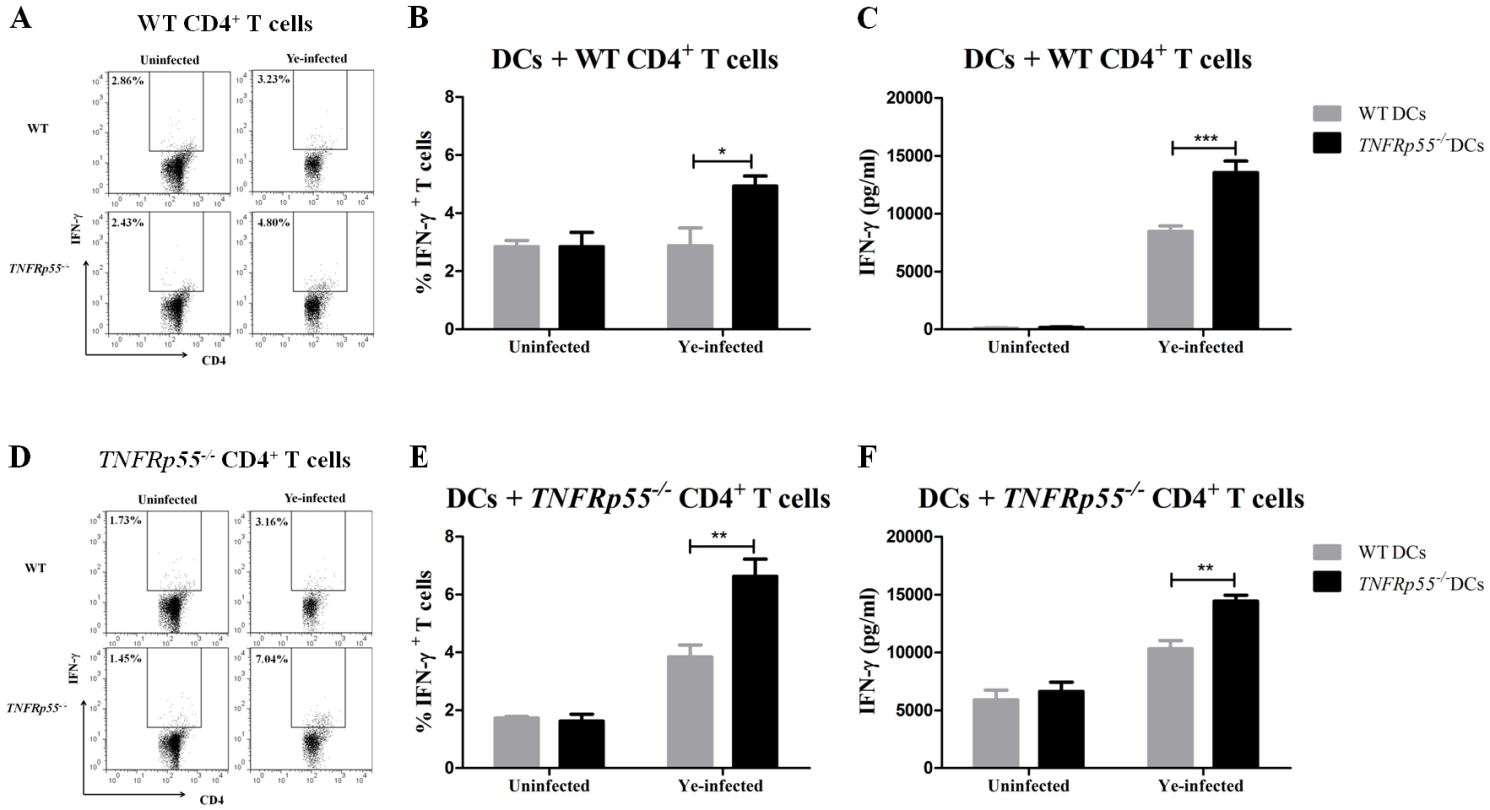
Th1 differentiation of CD4^+^ lymphocytes by WT or *TNFRp55*^*-/-*^DCs. Ye-infected splenic DCs of WT or *TNFRp55*^*-/-*^were co-cultured with purified CD4^+^ lymphocytes of Ye-infected WT (A), *TNFRp55*^*-/-*^mice (D). The cells were stained with cell surface CD4 and intracellular IFN-γ, and analyzed by flow cytometry. Dot plots from CD3^+^ CD4^+^gate, represent a sample of the statistical analysis shown in B and E (A and D). Percentages of WT CD4^+^ IFN-γ^+^ (B) and *TNFRp55*^*-/-*^ CD4^+^ IFN-γ^+^ (E) T cells. Levels of IFN-γ measured in culture supernatants by ELISA(C and F). Results are expressed as the mean ± SEM of quadruplets of one out of two independent experiments with four mice per group. *P<0.05; ** *P*<0.01; *** *P*<0.001.

**Fig 5:**
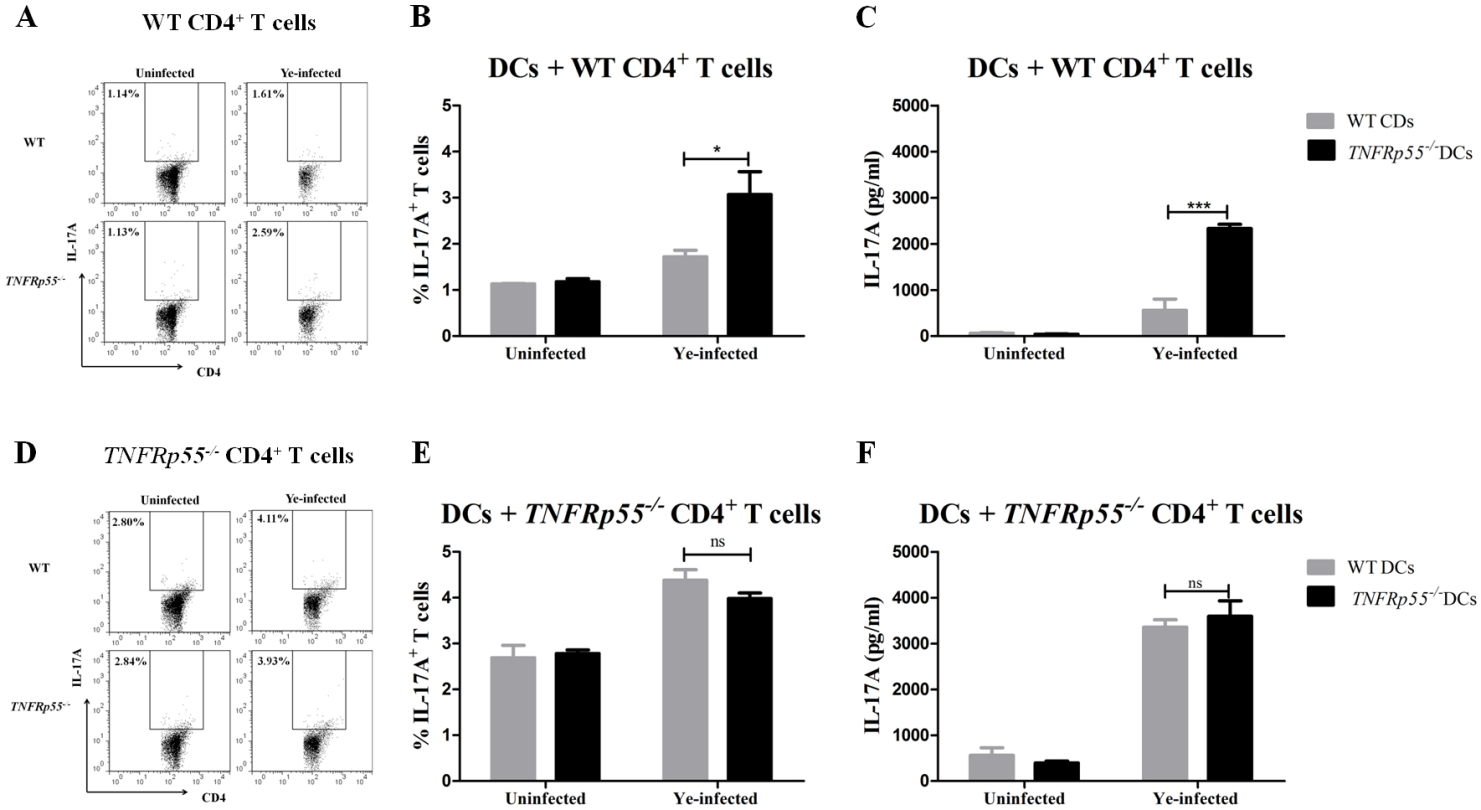
Th17 differentiation of CD4^+^ lymphocytes by WT or *TNFRp55*^*-/-*^ DCs. Ye-infected splenic DCs of WT or *TNFRp55*^*-/-*^were co-cultured with purified CD4^+^ lymphocytes of Ye-infected WT (A) or *TNFRp55*^*-/-*^mice (D) as described in methods. The cells were stained with cell surface CD4 and intracellular IL-17, and analyzed by flow cytometry. Dot plots from CD3^+^ CD4^+^ gate, represent a sample of the statistical analysis shown in B and E (A and D). Percentages of WT CD4^+^ IL-17A^+^ (B) and *TNFRp55*^*-/-*^ CD4^+^ IL-17A^+^ (E) T cells. Levels of IL-17A measured in culture supernatants by ELISA (C and F). Results are expressed as the mean ± SEM of quadruplets of one out of two independent experiments with four mice per group. *P<0.05; *** *P*<0.001. ns: not significant.

Ye-infected *TNFRp55*^*-/-*^ DCs favored the *in vitro* differentiation of WT CD4^+^ lymphocytes to Th1 (Fig 4A and 4B) and Th17 (Fig 5A and 5B) cells more than the Ye-infected WT DCs. In the same line, we detected significant amounts of IFN-γ (Fig 4C) and IL-17 (Fig 5C) in the supernatants of co-culture experiments of Ye-infected *TNFRp55*^*-/-*^ DCs with WT CD4^+^ lymphocytes. Moreover, Ye-infected *TNFRp55*^*-/-*^ DCs increased the frequency of Th1 (Fig 4E, S3 and S4 Figs and S1 Appendix) as well as IFN-γ secretion (Fig 4F, S1 Appendix) of *TNFRp55*^*-/-*^ CD4^+^ lymphocytes. Conversely, *TNFRp55*^*-/-*^ CD4^+^ lymphocytes did not increase neither on the frequency of Th17 nor on the production of IL-17 when they were co-cultured with Ye-infected WT or *TNFRp55*^*-/-*^ DCs (Fig 5E and 5F). These results indicate that the absence of TNFRp55 compromises DCs functions, facilitating the inflammatory Th1 and Th17 profiles, both previously reported in the joint of *TNFRp55*^*-/-*^mice that developed Ye-Induced ReA [23].

### Participation of MAPKs on IL-12/23p40 overproduction by *TNFRp55*^*-/-*^ DCs

**Fig 6:**
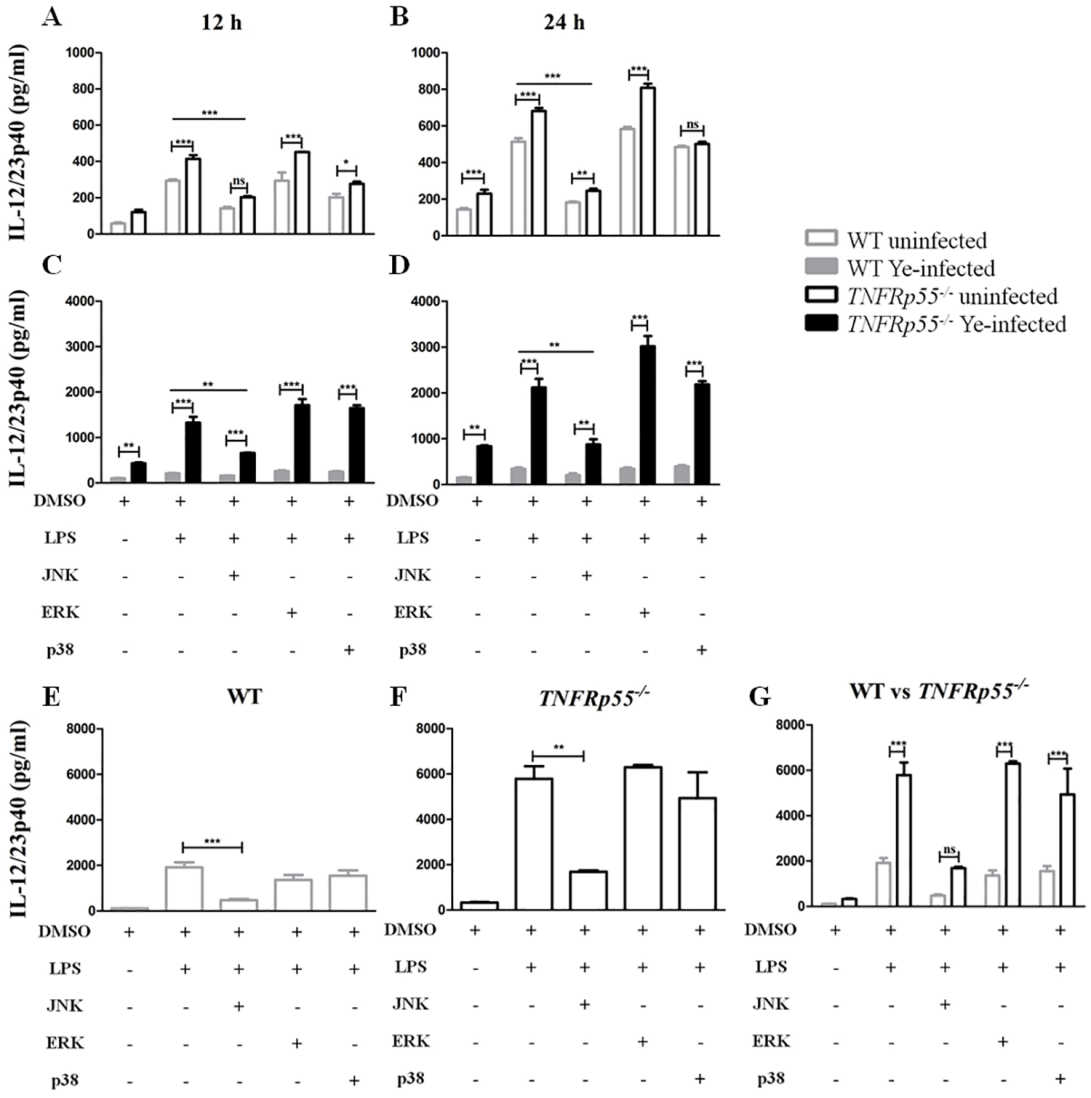
Participation of MAPKs on IL-12/23p40 overproduction by dendritic cells. Splenic cells were obtained from uninfected (A and B) and Ye-infected WT and *TNFRp55*^*-/-*^ mice (on ReA onset) (C and D), and stimulated *in vitro* with LPS in the presence of the indicated MAPK inhibitors. After 12 and 24h, supernatants were collected from the cell culture, and assayed by ELISA for IL-12/23p40. The same experimental scheme was performed with *in vivo* expanded and isolated WT and *TNFRp55*^*-/-*^ DCs. After 24h, IL-12/23p40 was assessed in the 24h-supernatants of WT (E) and *TNFRp55*^*-/-*^ (F) DCs. A comparison of IL-12/23p40 levels in the supernatants of WT versus *TNFRp55*^*-/-*^ DCs was performed (G). Results are expressed as the mean ± SEM of quadruplets of one out of two independent experiments with four mice per group. ** *P*<0.01; *** *P*<0.001. ns: not significant.

Next, we explored the intracellular signal transduction pathways responsible for higher IL-12/23p40 production by *TNFRp55*^*-/-*^ DCs when compared with WT DCs. One of the major inflammatory signaling pathways from the cell surface to the nucleus is the mitogen-activated protein kinase (MAPK) signal transduction pathway [33]. MAPKs belong to a large family of serine/threonine kinases, and include three well-characterized subfamilies: the c-Jun NH_2_-terminal kinases (JNK), the extracellular signal-regulated kinases (ERK) and the p38 family of kinases (p38 MAPKs) [34]. Therefore, these pathways were analyzed in splenocytes of uninfected (Fig 6A and 6B) and infected WT or *TNFRp55*^*-/-*^ mice obtained on day 14 (ReA onset) (Fig 6C and 6D). These cells were stimulated with LPS in the presence of JNK, ERK or p38 inhibitors. We found that splenocytes from uninfected *TNFRp55*^*-/-*^ mice significantly decreased the IL-12/23p40 production when JNK MAPK was inhibited (Fig 6A and 6B). This production reached the IL-12/23p40 levels secreted by WT cells at 12h or 24h of LPS-stimulation in the presence of JNK or p38 inhibitor, respectively (Fig 6A and 6B). Although significant reduction in the IL-12/23p40 production by infected *TNFRp55*^*-/-*^ splenocytes was achieved by JNK inhibition, IL-12/23p40 levels were still significantly higher than those secreted by WT splenocytes (Fig 6C and 6D). In good agreement with uninfected splenocytes, JNK inhibition also decreased the IL-12/23p40 secretion by splenic WT (Fig 6E) and *TNFRp55*^*-/-*^ (Fig 6F) DCs, reaching similar low levels (Fig 6G). Thus, JNK largely participated in the overproduction of IL-12/23p40 by *TNFRp55*^*-/-*^ DCs, while p38 MAPKs displayed a partial participation.

### IL-12/23p40 modulation by TNF in JAWSII cell line

**Fig 7:**
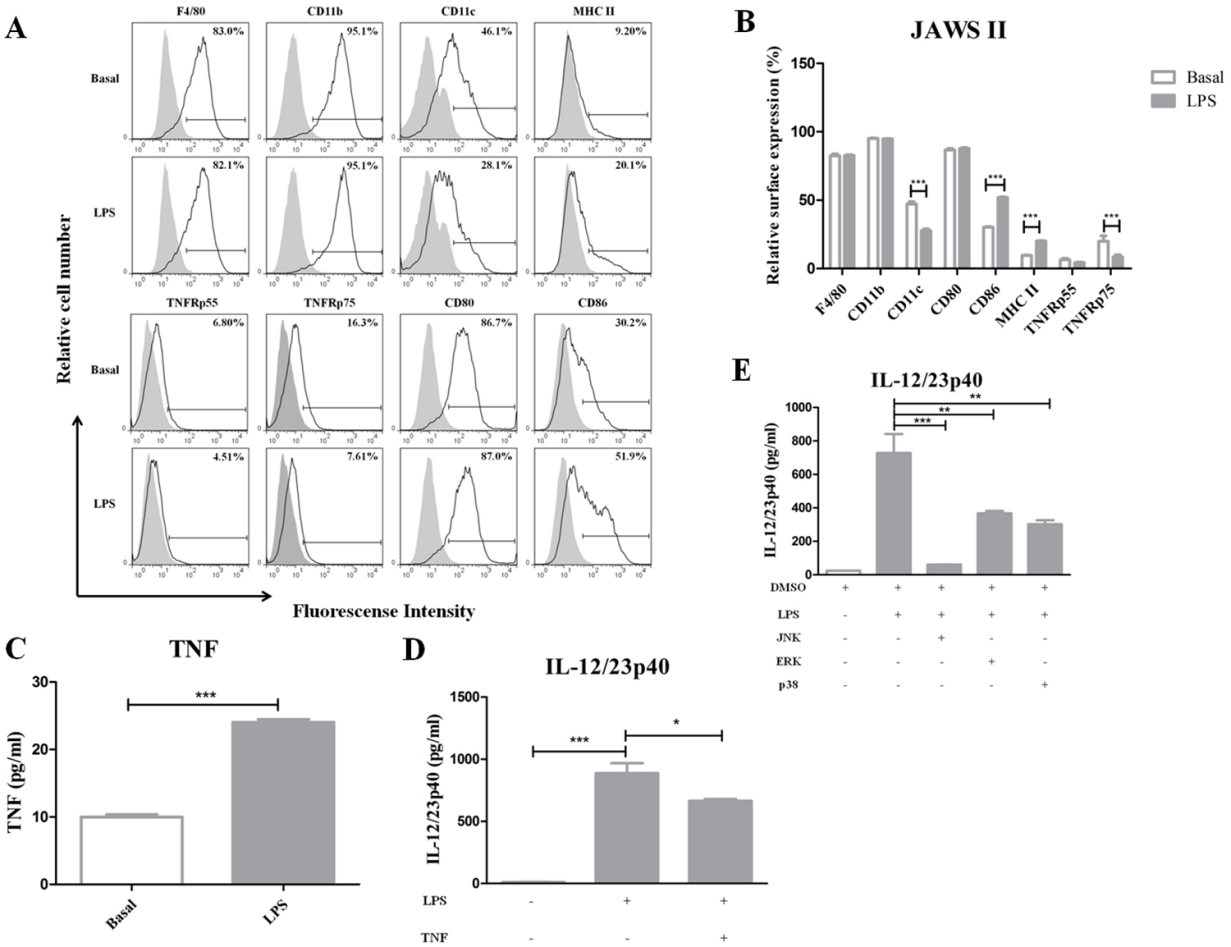
Phenotypical and functional characterization of JAWS II cell line. Representative overlaid flow cytometry histogram (A) and percentage (B) analysis, showing dendritic cell surface markers and TNF receptors in JAWS II cell line after 24h of stimulation with LPS. Unstimulated cells (basal) were used as controls. The levels of TNF (C) were assessed in the supernatants by ELISA. JAWS II cells were stimulated with LPS alone or in combination with human TNF (D), or in the presence of MAPK inhibitors (E). IL-12/23p40 secretion was analyzed in these supernatants. The results represent the mean ± SEM of quadruplets of one out of two independent experiments. *P<0.05; ** *P*<0.01; *** *P*<0.001.

JAWSII is an immortalized DC line derived from the bone marrow of *p53*^*-/-*^ mice [31]. We corroborated that these cells express surface markers of DCs, and that after LPS stimulation, they showed maturation increasing MHC-II and CD86 expressions, and decreasing CD11c marker (Fig 7A and 7B and S6 Fig). Interestingly, these cells expressed both TNFRp55 and TNFRp75 in moderate levels (Fig 7A and 7B and S6 Fig). In addition, the JAWSII cell line secreted TNF and IL-12/23p40 in response to LPS (Fig 7C and 7D). Human TNF (hTNF) has previously demonstrated to bind to mouse TNFRp55 but not to TNFRp75 [35]. Because of this, JAWS II cells were stimulated with LPS in the presence of hTNF, and a significant reduction of IL-12/23p40 secretion was observed (Fig 7D). Moreover, in JAWS II cell line the IL-12/23p40 production decreased significantly by p38, ERK and mainly JNK inhibitions (Fig 7E). As a consequence, these results suggest that JAWSII cells are suitable as a model to gain an insight into the mechanisms underlying the regulatory effect of TNF through TNFRp55 on IL-12/23p40 secretion, mirroring the results obtained in splenic DCs.

### Regulation of IL-12/23p40 production by TNFRp55 signaling and role of IL-10

**Fig 8:**
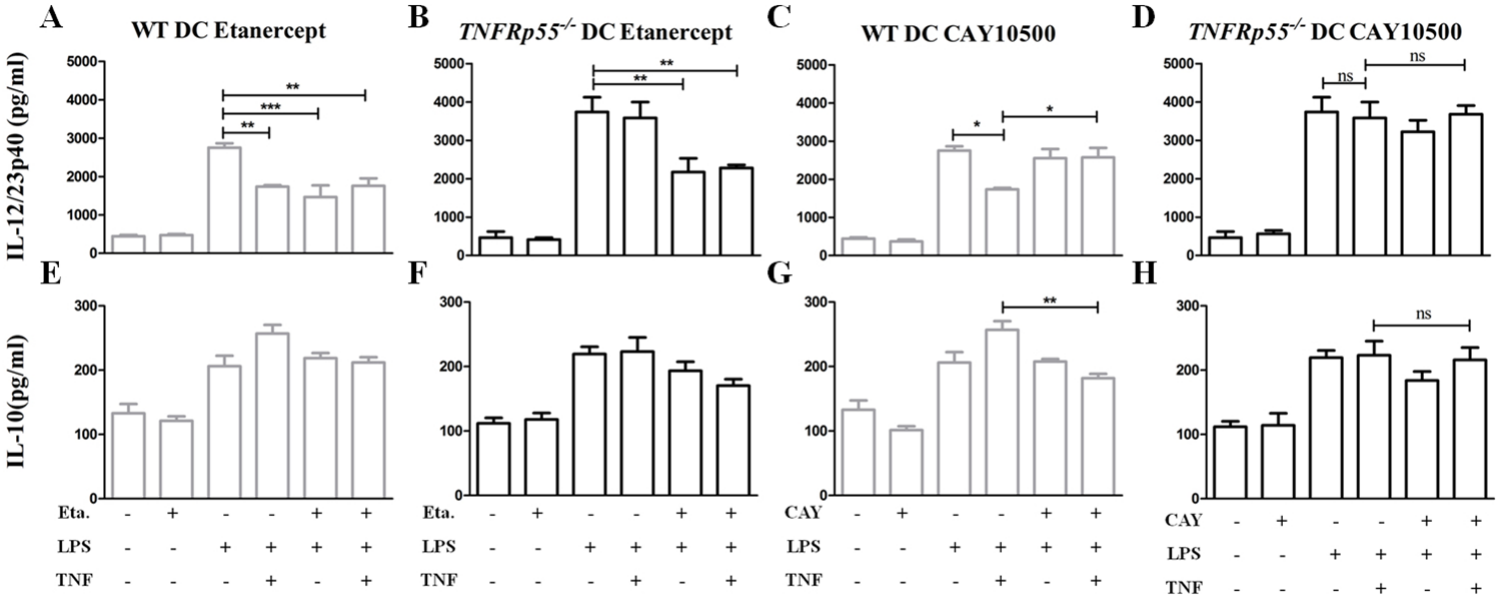
Contribution of TNFRp75 and TNFRp55 to IL-12/23p40 production by WT and *TNFRp55*^*-/-*^ dendritic cells. Isolated WT (A, C, E and G) and *TNFRp55*^*-/-*^ (B, D, F and H) DCs were stimulated with LPS alone or in the presence of Etanercept (A, B, E, F) that inhibits both TNFRp75 and TNFRp55; or CAY10500 (C, D, G and H) that blocks specifically TNFRp55. After 24h, the supernatants were collected and assayed by ELISA for IL-12/23p40 (A-D), and IL-10 (E-H). Results are expressed as the mean ± SEM of quadruplets of one out of two independent experiments. *P<0.05; ** *P*<0.01. ns: not significant.

In order to know how TNFRp55 signaling modulates IL-12/23p40 production, we isolated splenic WT or *TNFRp55*^*-/-*^ DCs, and stimulated with LPS in presence and absence of TNF or TNF inhibitors. We used Etanercept, which is a fusion protein consisting of the Fc domain of human IgG1 fused to a dimmer of the extracellular ligand-binding domain of human TNFRp75. It binds with high affinity to both soluble and transmembrane forms of TNF [36]. First, hTNF showed a regulatory effect on IL-12/23p40 secretion by LPS-stimulated WT DCs, and this effect was lost in *TNFRp55*^*-/-*^ DCs (Fig 8A and 8B). Moreover, Etanercept decreased IL-12/23p40 secretion in both LPS-stimulated WT and *TNFRp55*^*-/-*^DCs (Fig 8A and 8B). Furthermore, this TNF inhibitor did not change the TNF-mediated regulatory effect on IL-12/23p40 in WT DCs (Fig 8A). These results suggested a TNFRp75-depending IL-12/23p40 production by DCs, and a regulatory function of the TNFRp55 pathway.

To confirm these findings, DCs were stimulated with LPS in presence of CAY10500 inhibitor, which blocks specifically the TNF-TNFRp55 pathway[37]. In addition, we analyzed IL-10 levels in the supernatants, since this cytokine is one of the most important and known negative-regulators of IL-12/23p40 secretion [38]. As expected, CAY10500 inhibitor did not affect IL-12/23p40 induction by LPS. However, this TNFRp55-specific inhibitor blocked the regulatory effect of hTNFin WT DCs (Fig 8C). This result mirrored the effect of hTNF on LPS-stimulated *TNFRp55*^*-/-*^ DCs (Fig 8D). Conversely, IL-10 amount was not modified by Etanercept in LPS-stimulated WT as well as *TNFRp55*^*-/-*^ DCs (Fig 8E and 8F). However, IL-10 levels resulted significantly reduced by the CAY10500 inhibitor in hTNF/LPS-stimulated WT DCs, but not in *TNFRp55*^*-/-*^ DCs (Fig 8G and 8H). These data indicate that IL-10 seems to mediate the regulatory effect of the TNFRp55 pathway on IL-12/23p40 secretion by LPS-stimulated DCs.

## Discussion

DCs are well established as the most effective among the APC [32]. Strong efforts were made to elucidate the function of DCs in arthropathies such as rheumatoid arthritis [28], but little is known about their roles in ReA. In previous works, we demonstrated chronic ReA in *TNFRp55*^*-/-*^ mice after Ye infection [22], and pro-inflammatory Th1 and Th17 cells acting in concert to sustain this arthritogenic processes [23]. In line with this, an increased amount of IL-12/23p40, IL-12p70 and IL-23 in the joint draining lymph nodes provided a rational explanation for T helper deviation to Th1/Th17 responses [23,25]. Because of DCs ability to guide the Th differentiation by secretion of specific cytokines, this study was aimed to investigate the role of DCs on IL-12/23p40 overproduction inYe-induced ReA developed in *TNFRp55*^*-/-*^ mice.

TNF is a pleiotropic cytokine with a diverse range of biological activities. Further evidence points to the pro-inflammatory effects of TNF. Thus, this cytokine has been placed at the center of the complex scenario of the pathogenesis of immune-mediated inflammatory diseases, such as rheumatoid arthritis, ankylosing spondylitis and Crohn’s disease. It has also been confirmed by the efficacy of anti-TNF treatments [39–41].

However, there have been several investigations suggesting an anti-inflammatory role of TNF in limiting inflammatory processes induced by *in vivo* infections as well as by autoimmune diseases [42,43]. In addition, exacerbations of autoimmune diseases under anti-TNF therapy were attributed to anti-inflammatory effects of TNF [44]. Here, we found that splenic cells obtained from *TNFRp55*^*-/-*^ mice on ReA onset were unable to control secretions of pro-inflammatory cytokines in response to LPS. These findings support previous *in vitro* studies that recognized TNFRp55 as the main receptor involved in IL-12/23p40 regulation [29,39]. As reported by other authors, we detected that WT splenocytes obtained under inflammatory conditions were more susceptible to IL-12/23p40 regulation than those obtained under resting conditions [29]. In line with the known function of IL-12 to mediate Th1 response, we found a clear association between the significant increases of IL-12/23p40 and IFN-γproductions by *TNFRp55*^*-/-*^splenocytes [45]. In contrast, we did not detect direct connection between IL-12/23p40 and IL-17 productions. Moreover, a considerable IL-17 amount was secreted by infected WT and *TNFRp55*^*-/-*^ splenocytes in response to LPS. These findings might be due to the fact that Th17 differentiation is influenced not only by IL-23, but also by TGF-ß, IL-6 and IL-21[46].

Evidences in animal models implicate DCs in the SpA pathogenesis, a group of inflammatory arthropathies that include ReA [47]. It has been demonstrated that IL-23 mediates enthesitis, a hallmark of SpA-like disease, and that IL-23 was produced by CD11b^+^ population, which included macrophages and DCs [47]. In this study, we detected that TNFRp55 deficiency did not affect DC number in spleen, but impacted on the frequency of IL-12/23p40^+^DCs. These results indicated that DCs seem to be the cellular source of the IL-12/23p40 overproduction by *TNFRp55*^*-/-*^splenocytes. After *in vivo* expansion, we showed that isolated *TNFRp55*^*-/-*^ DCs secreted a larger amount of IL-12/23p40, and they could lead lymphocytes to Th1 and Th17 responses. Sing et al found higher levels of IL-17, IFN-γ and IL-12p40 in synovial fluids of patients with ReA/uSpA as compared to those with osteoarthritis [48]. Furthermore, it has been reported that Th17 are expanded and induced by DCs in SpA-prone HLA-B27-transgenic rats [49]. Therefore, IL-12/23p40 overproduction might be a mechanism used by DCs to drive Ye-induced ReA in *TNFRp55*^*-/-*^ mice.

Since it has been demonstrated that IL-12 is induced by LPS among other TLR ligands, we used LPS as TLR4 ligand for stimulating IL-12/23p40 in DCs. Furthermore, LPS represents a component of Gram-negative bacteria that mirrors *in vitro* a molecule present during *Yersinia* infection in our *in vivo* ReA model. Moreover, it has been also demonstrated that TNF is a potent regulator of IL-12p40 expression through TLR signaling pathways, including TLR4 [29,39]. In addition, the MAPKs are central downstream molecules in shuttling the signal of pro-inflammatory cytokines [50]. In our study, a considerable reduction on IL-12/23p40 production was detected in LPS-stimulated splenocytes and isolated DCs treated with JNK inhibitor. These findings indicate that JNK might mediate the IL-12/23p40 overproduction under TNFRp55deficiency. Indeed, JNK inhibition resulted in inhibition of TLR2-mediated IL-12/23p40 production [51]. In addition, it has been reported that JNK activates AP-1 transcription factor, which has a dual effect on IL-12/23p40 production, inducing [52] or regulating [53] mRNA transcription of this cytokine subunit. Here, the regulatory effect of TNF on IL-12/23p40 and the role of JNK on IL-12/23p40 were confirmed in the JAWS II cell line. Since a long time was necessary to obtain expanded DCs, we consider that this cell line will help in reducing time to uncover further downstream signaling involved in IL-12/23p40 regulation by TNFRp55.

**Fig 9:**
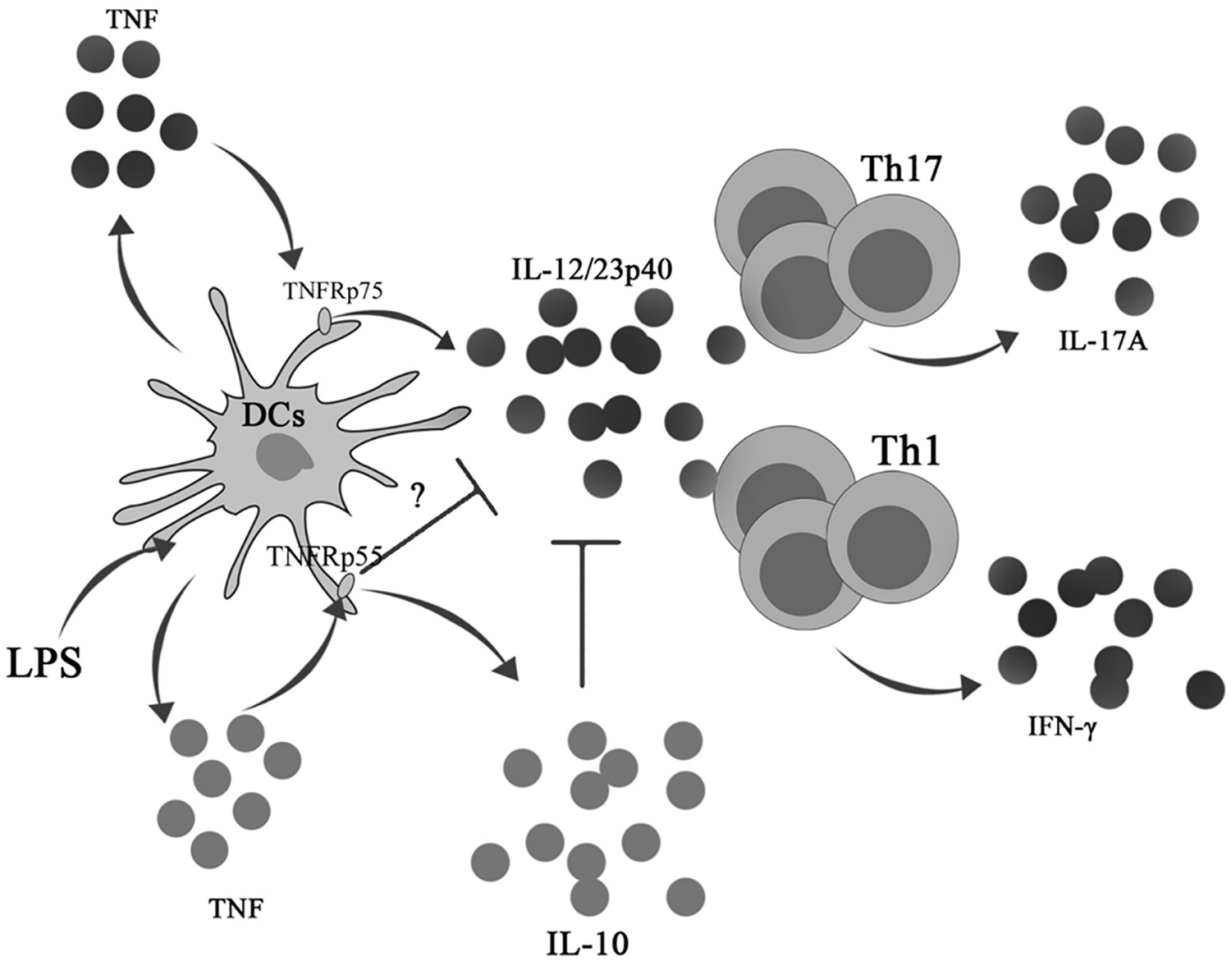
Proposal model of the participation of DCs controlling IL-12/23p40 through TNFRp55 and TNFRp75 under bacterial antigen stimulation. TNF through TNFRp75 triggers IL-12/23p40 secretion by DCs. This IL-12/23p40 production is controlled by TNF/TNFRp55 pathway involving IL-10 and other unknown mechanisms. In the absence of the regulatory TNF/TNFRp55 circuit, DCs increase IL-12/23p40 secretion, driving the adaptive response to chronic Th1 and Th17 programs. Black spots represent inflammatory cytokines, and grey spots represent regulatory cytokines.

The TNFRp55 signaling pathway includes the death domain-containing proteins (TNFR1, TRADD, RIP and FADD), leading to activation of signaling cascades that mediate JNK activation, and nuclear factor kappa-B (NF-κB) activation [20]. In addition, TLR4 plays important roles in inflammation by regulating the activity of NF-κB [54,55]. It has been shown that TNFRp55 and TLR-4 upstream signaling components are mostly receptor-specific, but the principles of signaling are similar, involving the recruitment of specific adaptor proteins and the activation of kinase cascades in which protein-protein interactions are controlled by poly-ubiquitination [54]. Moreover, these pathways include highly regulated proteins involving negative regulators that participate to induce an anti-inflammation response to a coordinated control feedback [54,55]. In this study, it was observed that Etanercept reduced IL-12/23p40 secretion in both LPS-stimulated WT and *TNFRp55*^*-/-*^ DCs, suggesting that TNFRp75, but not TNFRp55, participates in IL-12/23p40 production in our experimental conditions. In contrast, a specific inhibition of TNFRp55 signaling avoided the regulatory effect of TNF on IL-12/23p40 production by LPS-stimulated WT DCs. Therefore, it is unlikely that TNFRp75 signal plays a function in the anti-inflammatory effect of TNF. Rather, the data strongly support the role of the TNFRp55 pathway in keeping IL-12/23p40 under control. In addition, the results in infected *TNFRp55*^*-/-*^ splenocytes and LPS-stimulated DCs supported the involvement of IL-10 as a well-studied negative regulator of IL-12 secretion [37]. Furthermore, IL-10 regulates IL-12/23p40 production by transcriptional and epigenetic mechanisms [38]. However, we could not exclude other IL-10-independent mechanisms participating in the regulatory effect of TNF through TNFRp55, as suggested by other reports [44]. Because of this, we propose a model of DC role mediated by TNFRp55, in the control of Ye-induced ReA (Fig 9). Whether TNF/TNFRp55 pathway may interrupt directly TLR4 signaling has not been explored in our study. A previous report identified candidate proteins associated with a cross-talk between TNFRp55 and TLR4 signaling pathways [54], and indicated A20 as a negative regulator of the core crosstalk element TNFR-associated factor 6 (TRAF6), which has been demonstrated to be a main target of many regulatory molecules of TLR4 signaling [55].

Further studies will elucidate the intermediate molecules in the cross-talk among TNFRp55, IL-12/23p40 and IL-10 in DCs during the immune response against bacterial antigens.

In summary, we have shown that DCs are essential cells in the immunopathogenic scenario of Ye-induce ReA in *TNFRp55*^*-/-*^ mice. One mechanism of their participation is the overproduction of IL-12/23p40, leading to Th1 and Th17 programs. An anti-inflammatory function of the TNF/TNFRp55 pathway in DCs might contribute to the protection against ReA during the resolution of bacterial infection. Targeting this regulatory circuit might represent an option to control chronic arthritis.

## Supporting information

S1 Appendix Explanation of the two distinct CD3^+^CD4^+^ populations observed in S3 Fig. CD4 has an important role in Th response to antigen in the context of MHC-II molecules, initiating the events that lead to activation of these cells [*Ravichandran KS et al*, *Curr Top Microbiol Immunol*. *1996;205*:*47–62*]. In the co-culture experiments, we detected two distinct populations of CD4^+^ T cells (S3 Fig), one of them with lower CD4 expression. It has been demonstrated that under *in vitro* continuous stimulation, CD4 can be modulated on T cells [*Grishkan IV et al*, *Cell Immunol*. *2013; 284*: *68–74*]. Accordingly, in our work, CD4^+^ T cells received long *in vivo* and *in vitro* stimulation since they were obtained from the spleen of *TNFRp55*^*-/-*^ or WT mice on day 5 after Ye infection, and after enriching by magnetic beads, they were co-cultured with Ye-infected DCs for 5 days. During this time, we detected cellular proliferation of CD4^+^ T cells (S4 Fig). In accordance with the population that expressed lower levels of CD4 (gate 1 of S5 Fig), the percentage of proliferating cells was about 10% (S4 Fig), suggesting that these cells correspond to the CD4^+^ cells that expressed lower CD4. In addition, we observed that both CD4^+^ T cells populations displayed IFN-γ or IL-17 production, and they mirrored the differences observed in all CD4^+^ T cells (Figs 4 and 5 and S5 Fig). Therefore, we present in Figs 4 and 5 of the main manuscript the results of total CD4^+^ T cells that summery the response of the two CD4^+^ populations.
(DOCX)

S1 Fig Gating strategy for flow cytometry analysis of dendritic cells, and after intracellular IL-12/23p40 staining. A. In this sample gating, cells were first gated for leucocytes (SSC-H vs FSC-H) and then for dendritic cells (DCs) (CD11c^+^MHC-II^+^ gate). B. In this sample gating, cells were first analyzed as explained above and then DCs were further analyzed to measure IL-12/23p40 expression. Fluorescence-minus-one (FMO) control was included to define the selected population.
(TIF)

S2 Fig Gating strategy for flow cytometry analysis of isolated dendritic cells after *in vivo* expansion. Splenic CD11c^+^ cells were isolated by using anti-mouse CD11c magnetic beads. In this sample gating the isolated cells were gated for CD11c expression (SSC-H vs CD11c), and then for dendritic cells (DCs) (CD11c^+^MHC-II^+^ gate). CD80 and CD86 surface expression was then determined, and histogram analyses are shown. Fluorescence-minus-one (FMO) control (grey histogram) was included to define the selected population.
(TIF)

S3 Fig Gating strategy for flow cytometry analysis of CD4^+^ T cells used in co-culture assays. Splenic CD4^+^ cells were isolated by using anti-mouse CD4 magnetic beads. In this sample gating, cells were first gated for leucocytes (SSC-H vs FSC-H) and then for CD4^+^ T lymphocytes (CD3^+^ CD4^+^). Finally, the cells were further analyzed to measure IFN-γ or IL-17A expression. Fluorescence-minus-one (FMO) controls were used to define the selected population for each cytokine expression.
(TIF)

S4 Fig Proliferation of CD4^+^ T cells. CFSE-labeled WT or *TNFRp55*^*-/-*^ CD4^+^ T cells were co-cultivated at a 1:10 ratio with unlabeled WT or *TNFRp55*^*-/-*^ DCs (in Ye-infected or uninfected conditions). On day 5, the cells were collected and immediately analyzed using the FACSCalibur cytometer. A and C. Representative overlaid flow cytometry histogram analysis showing CFSE expression on lymphocytes based upon forward and side light scatter profiles. Numbers indicate percentages of proliferating CD4^+^cells. Unproliferating cells (grey histogram) were used to define the selected population. Percentages of WT CFSE^+^ CD4^+^ T cells (B) and *TNFRp55*^*-/-*^ CFSE^+^ CD4^+^ T cells (D). ns: not significant.
(TIF)

S5 Fig Gating strategy for flow cytometry analysis of IFN-γ or IL-17A expression by the two populations of CD4^+^ T cells in co-culture assays. A and F. In this sample gating, cells were first gated for leucocytes (SSC-H vs FSC-H) as showed in S3 Fig, then for CD4^+^ T lymphocytes (CD3^+^ CD4^+^), and finally two distinct populations were selected (gate 1 and gate 2). The cells of each gate were further analyzed to measure IFN-γ (A) or IL-17A (F) expression. *Fluorescence*-minus-one (FMO) controls were used to define the selected population for each cytokine expression. Percentages of WT CD4^+^ IFN-γ^+^ (B and C) and *TNFRp55*^*-/-*^ CD4^+^ IFN-γ^+^ (D and E) T cells. Percentages of WT CD4^+^ IL-17A^+^ (G and H) and *TNFRp55*^*-/-*^ CD4^+^ IL-17A^+^ (I and J) T cells. ** *P*<0.01; *** *P*<0.001. ns: not significant.
(TIF)

S6 Fig Gating strategy for flow cytometry analysis of JAWSII cell line. In this sample gating, cells were initially gated on SSC/FSC and then for live cells (7AAD negative). Finally, these cells were further analyzed to determine F4/80, CD11b, CD11c, MHC-II, CD80, CD86, TNFRp55 and TNFRp75 surface expression. Fluorescence-minus-one (FMO) control (grey histogram) was included to define the selected population in each histogram analysis.
(TIF)
