# Supplementary material for: IL-12/23p40 overproduction by dendritic cells leads to an increased Th1 and Th17 polarization in a model of Yersinia enterocolitica-induced reactive arthritis in TNFRp55-/- mice
Source: PLoS One. 2018 Mar 1;13(3):e0193573. doi: 10.1371/journal.pone.0193573 (PMC5832265; doi:10.1371/journal.pone.0193573)
Supplement: S1 Appendix — CD4 has an important role in Th response to antigen in the context of MHC-II molecules, initiating the events that lead to activation of these cells [Ravichandran KS et al, Curr Top Microbiol Immunol. 1996;205:47–62]. In the co-culture experiments, we detected two distinct populations of CD4+ T cells (S3 Fig), one of them with lower CD4 expression. It has been demonstrated that under in vitro continuous stimulation, CD4 can be modulated on T cells [Grishkan IV et al, Cell Immunol. 2013; 284: 68–74]. Accordingly, in our work, CD4+ T cells received long in vivo and in vitro stimulation since they were obtained from the spleen of TNFRp55-/- or WT mice on day 5 after Ye infection, and after enriching by magnetic beads, they were co-cultured with Ye-infected DCs for 5 days. During this time, we detected cellular proliferation of CD4+ T cells (S4 Fig). In accordance with the population that expressed lower levels of CD4 (gate 1 of S5 Fig), the percentage of proliferating cells was about 10% (S4 Fig), suggesting that these cells correspond to the CD4+ cells that expressed lower CD4. In addition, we observed that both CD4+ T cells populations displayed IFN-γ or IL-17 production, and they mirrored the differences observed in all CD4+ T cells (Figs 4 and 5 and S5 Fig). Therefore, we present in Figs 4 and 5 of the main manuscript the results of total CD4+ T cells that summery the response of the two CD4+ populations. (DOCX) [file pone.0193573.s001.docx]

**S1 Appendix**

CD4 has an important role in Th response to antigen in the context of MHC-II molecules, initiating the events that lead to activation of these cells [*Ravichandran KS et al, Curr Top Microbiol Immunol. 1996;205:47-62*]. In the co-culture experiments, we detected two distinct populations of CD4^+^ T cells (S3 Fig), one of them with lower CD4 expression. It has been demonstrated that under *in vitro* continuous stimulation, CD4 can be modulated on T cells [*Grishkan IV et al, Cell Immunol. 2013; 284: 68-74*]. Accordingly, in our work, CD4^+^ T cells received long *in vivo* and *in vitro* stimulation since they were obtained from the spleen of *TNFRp55^-/-^* or WT mice on day 5 after Ye infection, and after enriching by magnetic beads, they were co-cultured with Ye-infected DCs for 5 days. During this time, we detected cellular proliferation of CD4^+^ T cells (S4 Fig). In accordance with the population that expressed lower levels of CD4 (gate 1 of S5 Fig), the percentage of proliferating cells was about 10% (S4 Fig), suggesting that these cells correspond to the CD4^+^ cells that expressed lower CD4. In addition, we observed that both CD4^+^ T cells populations displayed IFN-γ or IL-17 production, and they mirrored the differences observed in all CD4^+^ T cells (Fig 4, Fig 5 and S5 Fig). Therefore, we present in Figures 4 and 5 of the main manuscript the results of total CD4^+^ T cells that summery the response of the two CD4^+^ populations.
