# Supplementary figures and images for: IL-12/23p40 overproduction by dendritic cells leads to an increased Th1 and Th17 polarization in a model of Yersinia enterocolitica-induced reactive arthritis in TNFRp55-/- mice
Source: PLoS One. 2018 Mar 1;13(3):e0193573. doi: 10.1371/journal.pone.0193573 (PMC5832265; doi:10.1371/journal.pone.0193573)

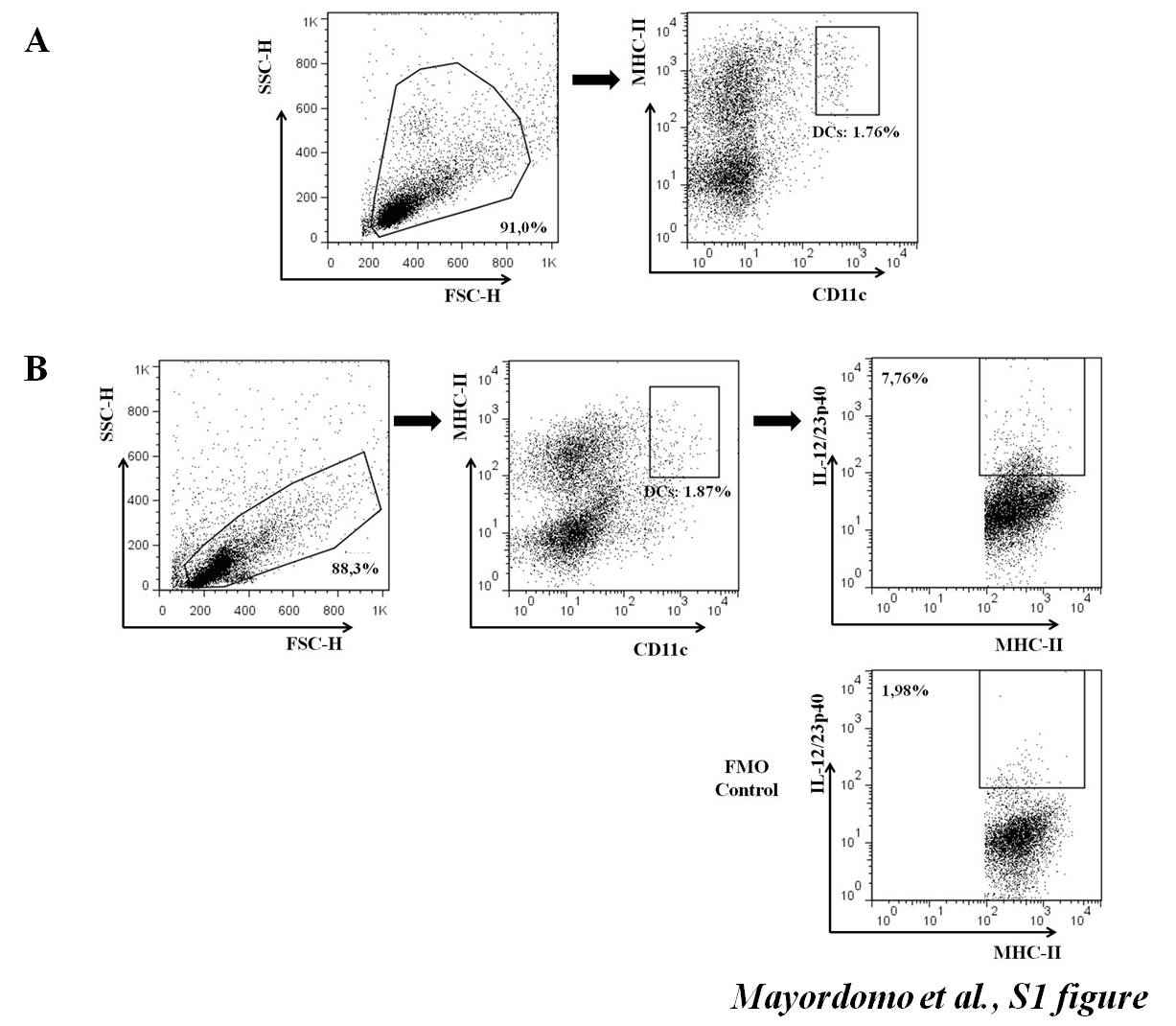

Supplement: S1 Fig — A. In this sample gating, cells were first gated for leucocytes (SSC-H vs FSC-H) and then for dendritic cells (DCs) (CD11c+MHC-II+ gate). B. In this sample gating, cells were first analyzed as explained above and then DCs were further analyzed to measure IL-12/23p40 expression. Fluorescence-minus-one (FMO) control was included to define the selected population. (TIF) [file pone.0193573.s002.tif]

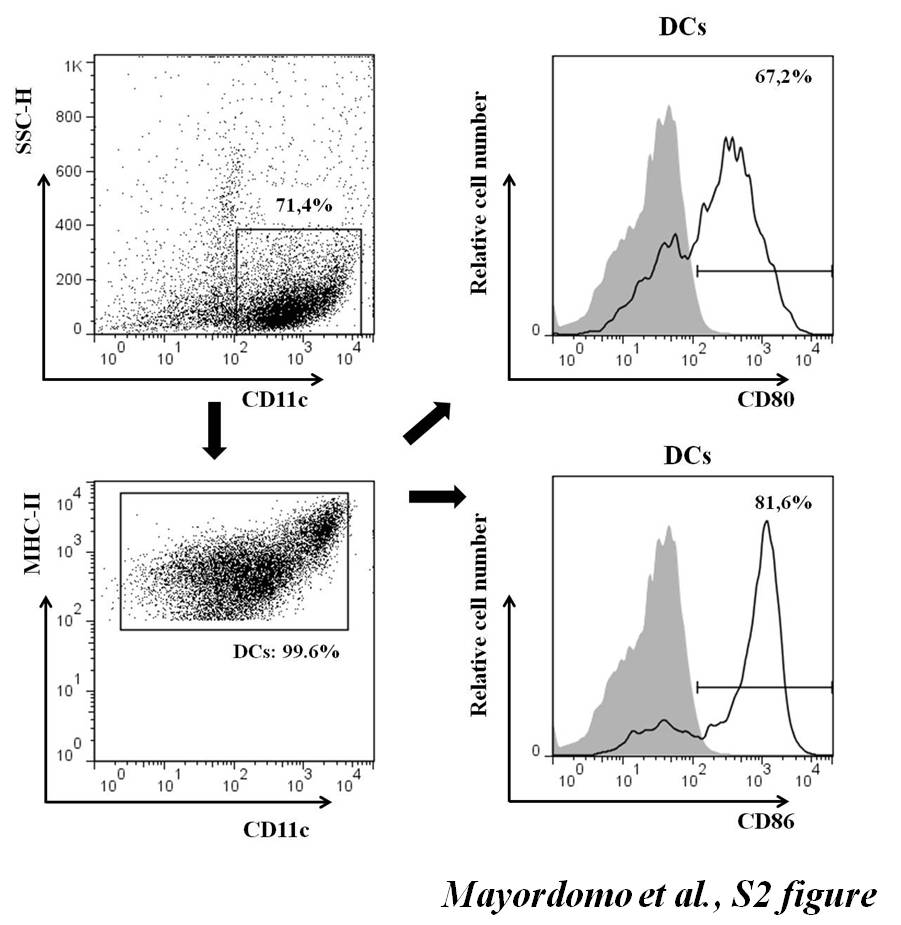

Supplement: S2 Fig — Splenic CD11c+ cells were isolated by using anti-mouse CD11c magnetic beads. In this sample gating the isolated cells were gated for CD11c expression (SSC-H vs CD11c), and then for dendritic cells (DCs) (CD11c+MHC-II+ gate). CD80 and CD86 surface expression was then determined, and histogram analyses are shown. Fluorescence-minus-one (FMO) control (grey histogram) was included to define the selected population. (TIF) [file pone.0193573.s003.tif]

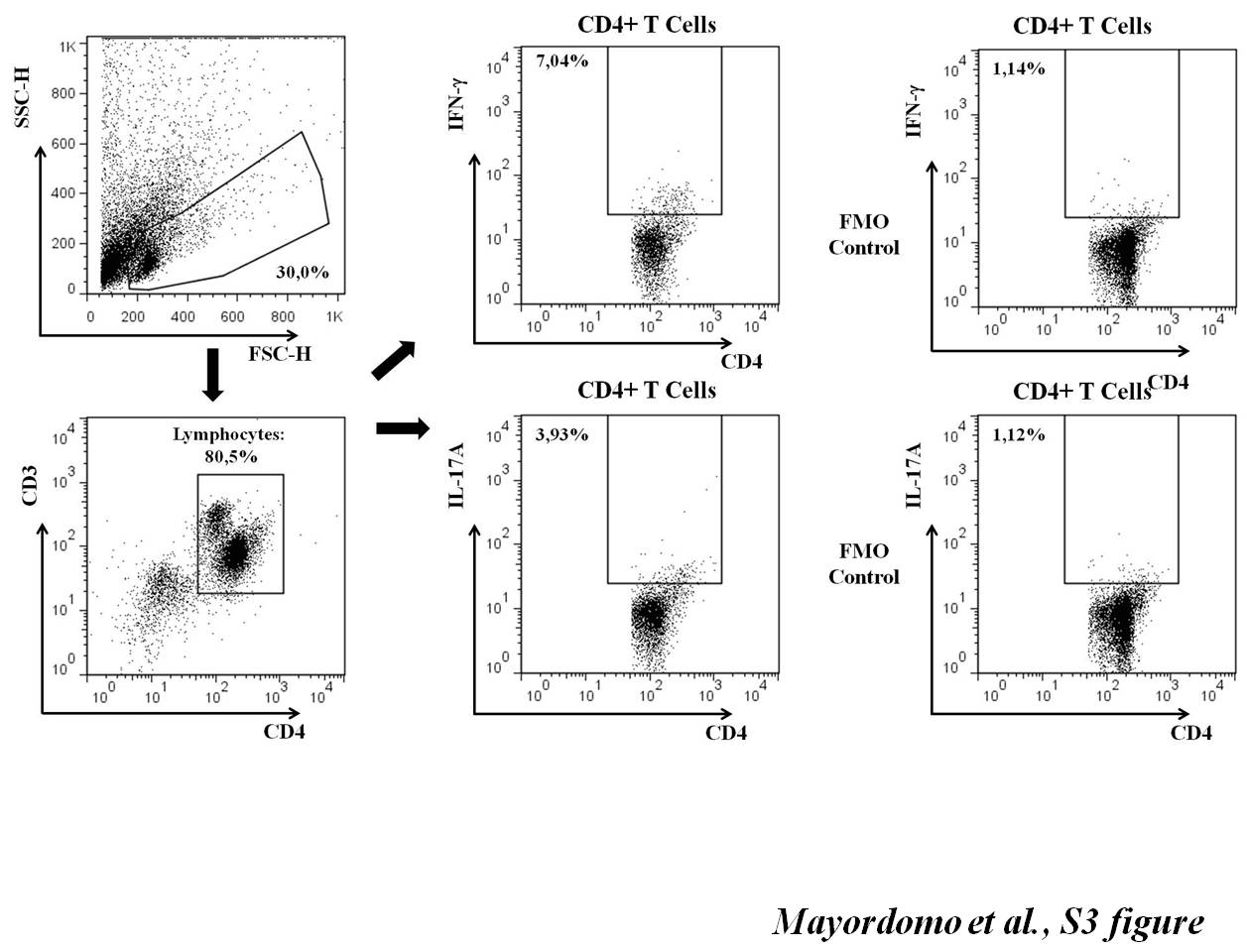

Supplement: S3 Fig — Splenic CD4+ cells were isolated by using anti-mouse CD4 magnetic beads. In this sample gating, cells were first gated for leucocytes (SSC-H vs FSC-H) and then for CD4+ T lymphocytes (CD3+ CD4+). Finally, the cells were further analyzed to measure IFN-γ or IL-17A expression. Fluorescence-minus-one (FMO) controls were used to define the selected population for each cytokine expression. (TIF) [file pone.0193573.s004.tif]

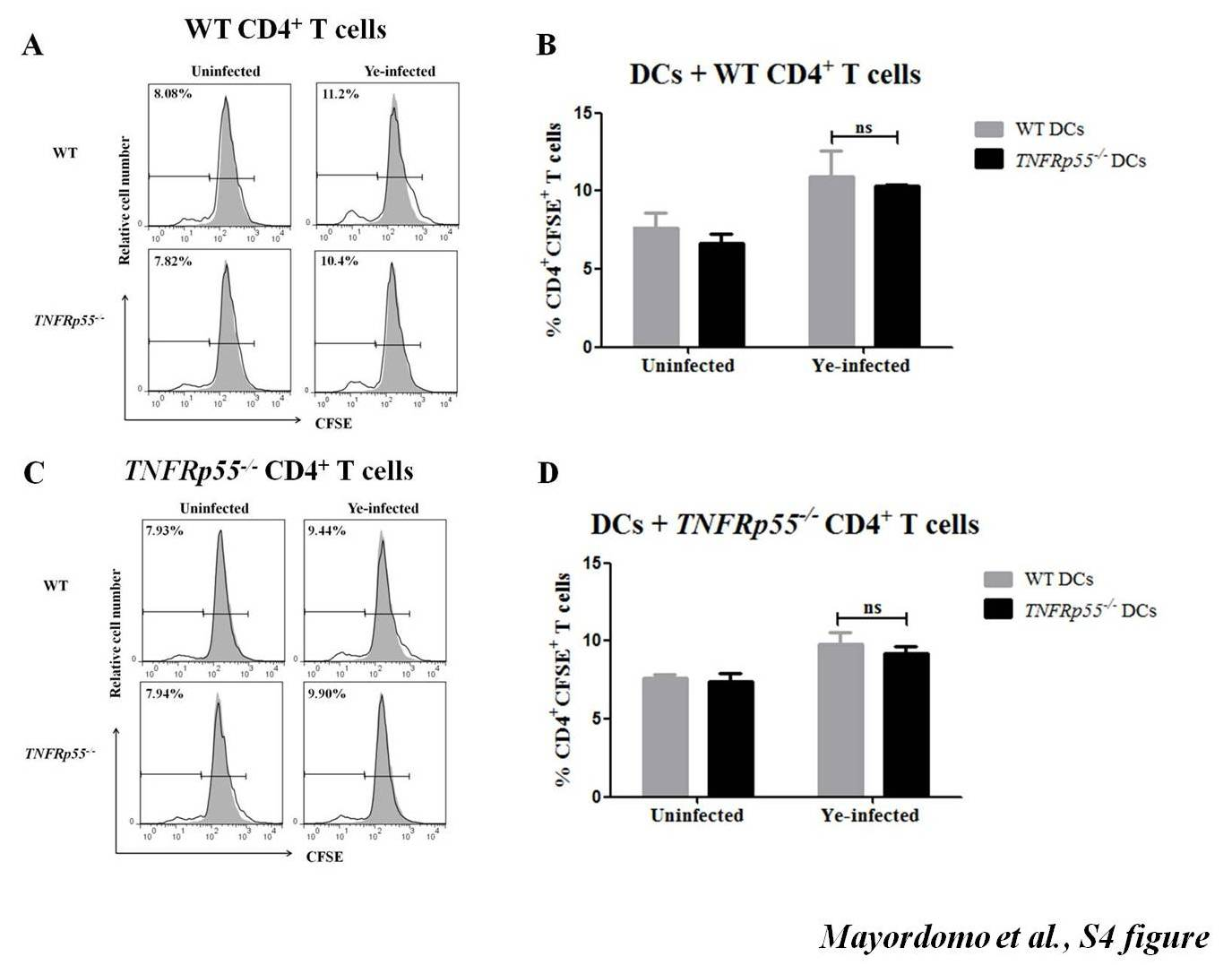

Supplement: S4 Fig — CFSE-labeled WT or TNFRp55-/- CD4+ T cells were co-cultivated at a 1:10 ratio with unlabeled WT or TNFRp55-/- DCs (in Ye-infected or uninfected conditions). On day 5, the cells were collected and immediately analyzed using the FACSCalibur cytometer. A and C. Representative overlaid flow cytometry histogram analysis showing CFSE expression on lymphocytes based upon forward and side light scatter profiles. Numbers indicate percentages of proliferating CD4+cells. Unproliferating cells (grey histogram) were used to define the selected population. Percentages of WT CFSE+ CD4+ T cells (B) and TNFRp55-/- CFSE+ CD4+ T cells (D). ns: not significant. (TIF) [file pone.0193573.s005.tif]

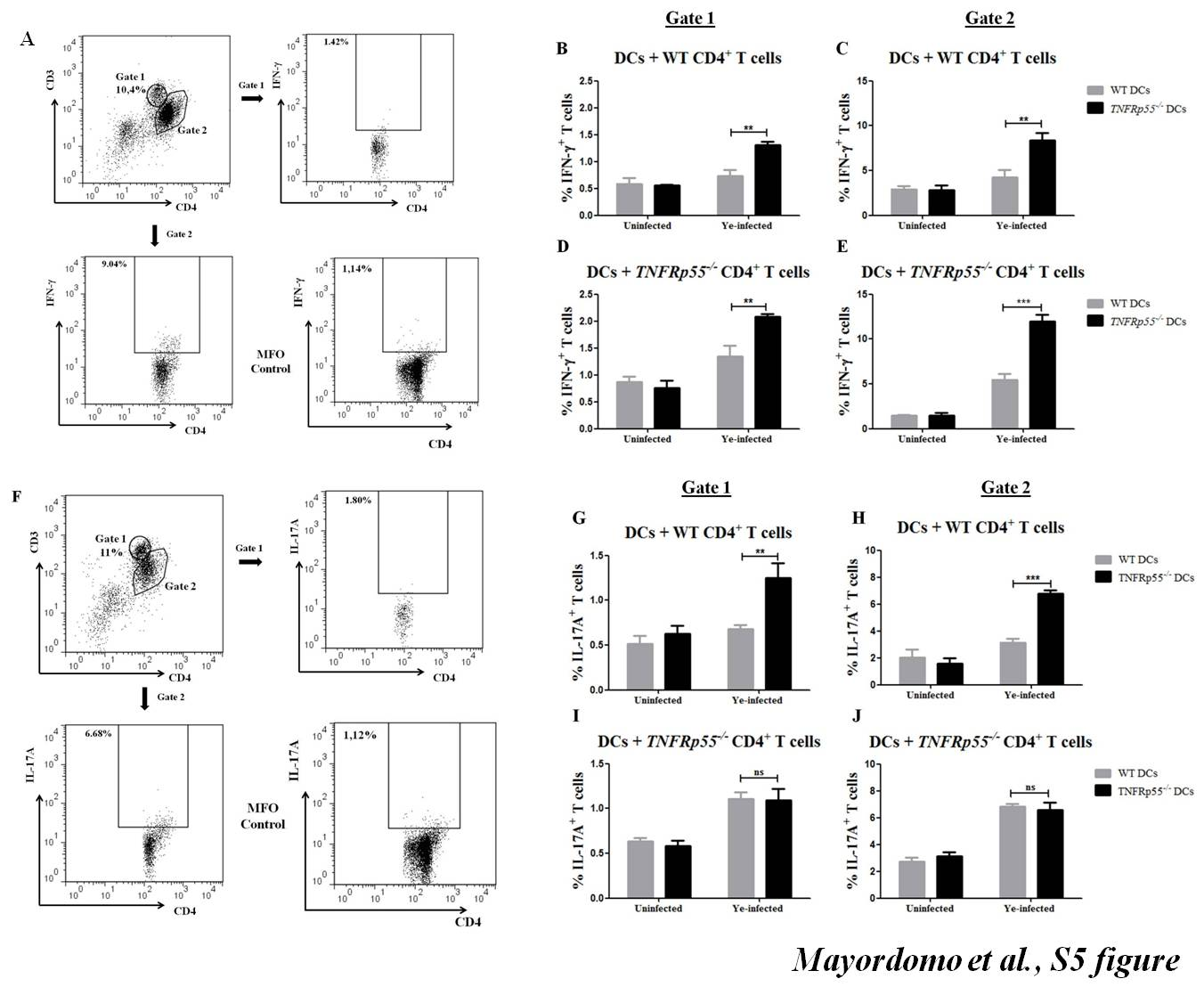

Supplement: S5 Fig — A and F. In this sample gating, cells were first gated for leucocytes (SSC-H vs FSC-H) as showed in S3 Fig, then for CD4+ T lymphocytes (CD3+ CD4+), and finally two distinct populations were selected (gate 1 and gate 2). The cells of each gate were further analyzed to measure IFN-γ (A) or IL-17A (F) expression. Fluorescence-minus-one (FMO) controls were used to define the selected population for each cytokine expression. Percentages of WT CD4+ IFN-γ+ (B and C) and TNFRp55-/- CD4+ IFN-γ+ (D and E) T cells. Percentages of WT CD4+ IL-17A+ (G and H) and TNFRp55-/- CD4+ IL-17A+ (I and J) T cells. ** P<0.01; *** P<0.001. ns: not significant. (TIF) [file pone.0193573.s006.tif]

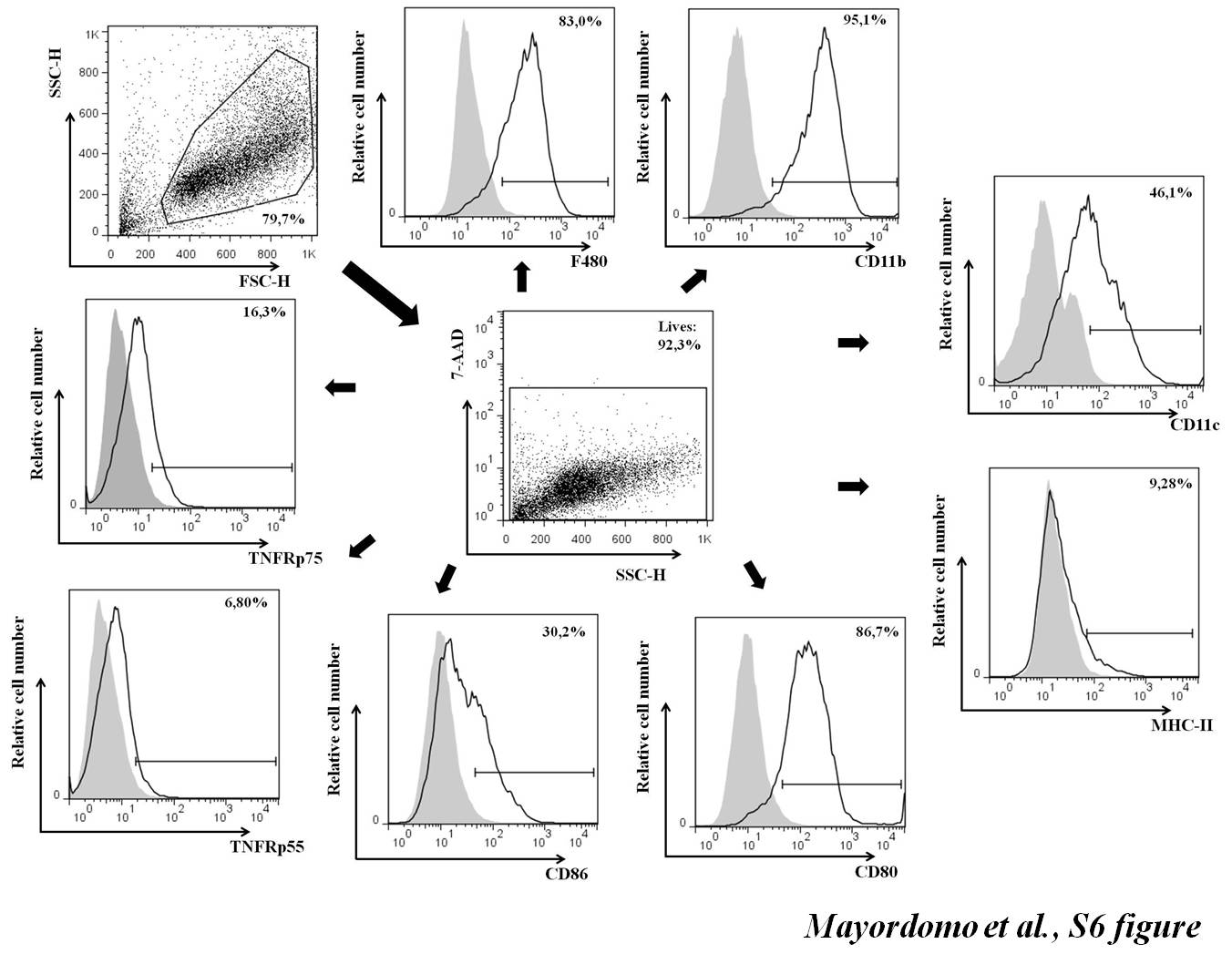

Supplement: S6 Fig — In this sample gating, cells were initially gated on SSC/FSC and then for live cells (7AAD negative). Finally, these cells were further analyzed to determine F4/80, CD11b, CD11c, MHC-II, CD80, CD86, TNFRp55 and TNFRp75 surface expression. Fluorescence-minus-one (FMO) control (grey histogram) was included to define the selected population in each histogram analysis. (TIF) [file pone.0193573.s007.tif]
